# Multiwall Rectangular Plates under Transverse Pressure—A Non-Linear Experimental and Numerical Study

**DOI:** 10.3390/ma16052041

**Published:** 2023-03-01

**Authors:** Gilad Hakim, Haim Abramovich

**Affiliations:** Faculty of Aerospace Engineering, Technion—Israel Institute of Technology (I.I.T.), Haifa 32000, Israel

**Keywords:** rectangular plate large deflection, Föppl–von Kármán equations, multiwall plate, nonlinear load–deflection curve, vacuum chamber loading test

## Abstract

Large deflection of rectangular plates under transverse pressure is described by Föppl–von Kármán equations, which have only approximated solutions. One of these methods is the separation into a small deflection plate and a thin membrane described by a simple third order polynomial expression. The present study presents an analysis to obtain analytical expressions for its coefficients by using the plate’s elastic properties and dimensions. To validate the non-linear relationship between the pressure and the lateral displacement of the multiwall plate, a vacuum chamber loading test is used to measure the plate’s response, with a large number of plates and length–width combinations. In addition, to further validate the analytical expressions, several finite element analyses (FEA) were performed. It has been found that the polynomial expression fairly describes the measured and calculated deflections. This method allows the prediction of plate deflections under pressure as soon as the elastic properties and the dimensions are known.

## 1. Introduction

The problem of large deflection of plates has attracted much attention since the end of the 19th century, due to its technical importance. Unfortunately, this problem has been found to be difficult to solve. The difficulties were raised at an early stage with Föppl equations for large deflections of membranes [1], where no closed-form solution was found for the rectangular membrane case. In 1910, this equation set was enhanced by Theodor von Kármán [2] to include the bending resistance of plates. 

The Föppl–von Kármán equation set has challenged many researchers over the years. Nevertheless, only approximated solutions were developed, most of which are rather difficult to implement.

It was August Föppl himself who suggested an approximate approach. This approach is mentioned in Timoshenko [3] (p. 423) footnote 1, which mentions Föppl’s “Drang und Zwang” [4] (p. 345). The approach is that the transverse distributed load *q* on the plate can be separated into two parts: *q* = *q*_1_ + *q*_2_. The first part *q*_1_ is balanced by the plate’s bending and shearing resistance, which are calculated through the plate small deflection linear theory. The second part *q*_2_ is balanced by the large deflections in-plane membrane forces only. Using the mid-point deflection *w*, this approximation is written as:

(1) $$q=q_{1}+q_{2}=A\cdot w+B\cdot w^{3}$$

The plate’s small deflection coefficient *A* has been calculated in many previous studies, with most of them using a summation of the Fourier series. The large deflection coefficient *B*, however, has no exact solution, as it is ascribed to the difficult Föppl’s membrane problem [1].

This expression (1) for the load–deflection behavior is quoted by many sources, such as Timoshenko [3] (p. 424) for square isotropic plates, Ugural [5] (p. 358), Wang and El-Sheikh [6] (p. 816), as well as many others.

One of them is Riber [7], who had suggested a “combined analytical solution” (see p. 71 in [7]) to find the constants in Equation (1). He used an energy method to obtain rather complex expressions for the coefficients *A* and *B* (see Equation (1)). He also presented simplified expressions for the constant *B* but with some internal inconsistencies. Riber [7] assumed in-plane immovable edges, which is not the case to be presented in the present study—see the BCs (Boundary Conditions) discussion later in the paper. Nevertheless, his *B* equation has inspired the presentation of a better expression for the coefficient *B* later in the present study.

Awrejcewicz et al. [8] present many efficient numerical methods that can be used to calculate specific rectangular plates without orthotropic and transverse shear behaviors.

Battaglia et al. [9] analyze orthotropic membranes and show that the load is proportional to $w^{3}$, where the proportion coefficient can be compared to our cases.

Maier-Schneider et al. [10] found the membrane proportion coefficient with both the improved analytic energy method and the finite element method with a good agreement.

Niyogi [11] solves the simply supported orthotropic plate problem with an approximate Galerkin–Bubnov procedure. The resulting *q* = *Aw* + *Bw*³ expression is verified for the isotropic plate only. The edge in-plane BCs are immovable.

Wang et al. [12] also solve the static behavior of flat glass plate and present load–deflection data graphically. No explicit mathematical expression is given for that, although it can be extracted from the graph. Here, the in-plane BCs are totally immovable.

References [13,14,15] provide further theoretical elasticity basis on the present investigated topic.

During the literature survey, several sources were found referring to tests and calculation methods of plates’ large deflection. In order to compare the results of these papers, it was necessary to normalize the various data to a common comparable structure. The structure was a thin square isotropic plate with movable edges and an evenly distributed transverse load. The coefficients of Equation (1), *A* and *B*, were calculated considering the plate dimensions and the material properties. The result of this comparison has shown the considerable variability of the coefficient *B*. This variability was unexpected since most of the data was based on real laboratory tests that should respond in a similar way. This may demonstrate the fact that it is not easy to correctly measure this property. A full description of the comparison with a suggested explanation is presented in Appendix B.

## 2. Multiwall Plates

The structure of multiwall plates consists of two thin face sheets separated by an internal structure of ribs and walls. The plate is usually produced by extrusion, in which a melted material is pressed through a die with the required shape. The materials used are aluminum and various plastics. The result is a thick, endless plate with a fixed cross-section shape along the extrusion direction and width according to the equipment size. The plate is then cut to the desired length.

**Figure 1:**
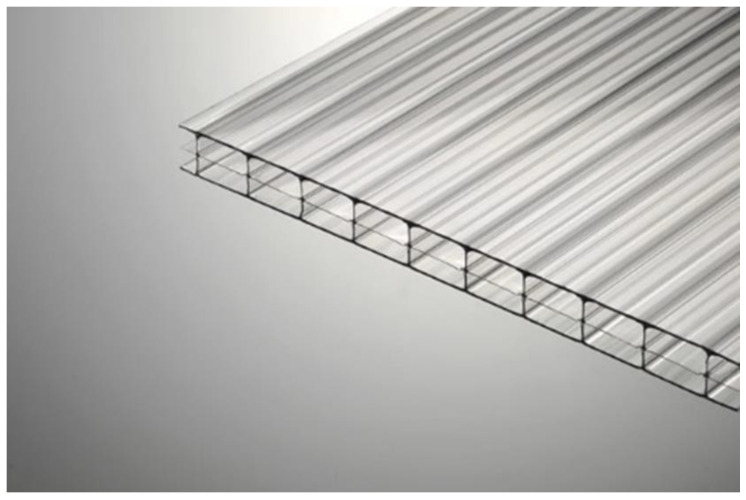
A 16 mm Multiwall PC Plate.

The plate is made of polycarbonate (PC), which is a tough transparent plastic. A typical 16mm PC plate can be seen in Figure 1.

The main application of PC multiwall plates is the glazing of architectural spaces, where both natural light and weather protection are required. As a result, the plates are exposed to wind and snow loads, which they must safely resist.

Currently, no publication describes the general performance of these plates, except manufacturer’s datasheets, which are very limited to specific products and specific applications. The available publications about latticed structures relate to specific shapes, such as triangles and trapezoids, and not a general approach as presented here, which can be considered novel.

The available approximated solutions for large deflections of plates are generally rather complicated, and in many cases, they involve a computational process, which is not straight-forward for field engineers. The non-linear nature of load–deflection curves is not easily represented in these solutions. Most of the research works already done do not cover the full complexity of the multiwall plates, which are shear, deformable, and orthotropic. Therefore, an engineer who needs to design a system with multiwall plates will probably face serious difficulties. This situation justifies this paper, which yields a first rough guess of these plates’ performance.

To calculate the multiwall plate response to distributed load, it is necessary to know the plate’s equivalent elastic properties, its dimensions (length–width), and the boundary conditions. Looking at the multiwall structure, it is obvious that the plate is orthotropic for both bending and tension, and its cross-section is transverse shear deformable. In the present study, it is assumed that all necessary equivalent elastic properties are already known. One should note that a procedure to obtain these equivalent properties of the plate is presented in Hakim and Abramovich [16].

## 3. Axes System

**Figure 2:**
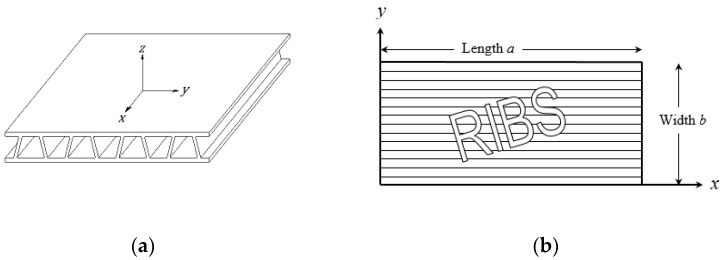
Axes directions: (**a**) 3D view trapezoid plate, (**b**) 2D view ribbed plate.

The axes directions are defined as shown in Figure 2, where axis *x* is the extrusion direction and *z* axis is normal to the plate surface:

The origin location of the axes may be set to any convenient place.

## 4. Boundary Conditions (BCs)

For small deflections analysis, the assumption is that all in-plane stress, strains, and deflections are negligible. Therefore, the BCs, here, ignore the in-plane conditions. The most commonly used BCs are: Free (F)-no restrictions, Simply Supported (S)-no *z* deflection but free rotation (no bending moments), and Clamped (C)-no *z* deflection and no rotations (zero slope). S and C are the two extremes of the more complicated BC-flexible rotation support, which is rarely used.

For large deflection analysis, the in-plane BCs must be considered. The two most common BCs are: Immovable (I)-the plate edge is fixed to the support and Movable (M)-the plate edges are allowed to move. It is necessary to specify both in-plane movement directions: normal to the edge and parallel to the edge. The BC used later here is SSSS-M, in which the four S stands for the four plate sides simply supported, and the M stands for the movable edges in both normal and parallel directions.

The movable (M) condition requires additional attention. When the Föppl’s approximation is used, the plate in a large deflection regime is a membrane. Its deflection on movable boundary conditions should then be calculated. However, a well-known property of a membrane is that it cannot sustain in-plane compression forces, as it immediately wrinkles. However, a real plate does resist compression, as it has a bending rigidity. We, therefore, have to analyze a membrane with movable edges, which may have compression stress. Mathematically, it is possible (with the known difficulties of Föppl equations), but other practical problems would appear. Since this transversely loaded M membrane is not a common case in the literature and, perhaps, even physically not possible, no previous scientific papers that would suggest a possible solution were found. Nevertheless, an expression is suggested later in this paper.

## 5. Methods

### 5.1. A-B Analytical Prediction

The expressions that would predict the values for the coefficients A and B are displayed next. The variables used are:

- a [m] Plate length
  b [m] Plate width
  h [m] Plate thickness
  q [Pa] Distributed load
  w [m] Mid-point deflection
  m, n  Summation indices

The plate equivalent properties are assumed to be known a priori:

- $D_{x}$ [Nm] Plate *x*-direction bending rigidity
  $D_{y}$ [Nm] Plate *y*-direction bending rigidity
  $D_{xy}$ [Nm] Plate twist rigidity
  $\nu_{x}^{b}$, $\nu_{y}^{b}$ Bending Poisson’s ratios
  $S_{x}$, $S_{y}$ [N/m] Transverse shear rigidity in *x* and *y* directions
  $E_{x}^{t}$, $E_{y}^{t}$ [Pa] Equivalent plate tension E moduli in *x* and *y* direction
  $\nu_{x}^{t}$, $\nu_{y}^{t}$ Tension Poisson’s ratios

The *x*, *y* axes origin is set as shown in Figure 2b—at the plate corner.

### 5.2. Small Deflection Coefficient A

The expression in (2) was derived using the Libove and Batdorf NACA Report No. 899 [17].

(2) $$w=\frac{q}{A}=\frac{16q}{\pi^{6}}\sum_{n=1,3,5\ldots }^{\infty }\sum_{m=1,3,5\ldots }^{\infty }\frac{\left[\begin{array}{l} \pi^{4}K_{8}{\left(\frac{m}{a}\right)}^{4}+\pi^{4}K_{9}{\left(\frac{m}{a}\right)}^{2}{\left(\frac{n}{b}\right)}^{2}+\pi^{4}K_{10}{\left(\frac{n}{b}\right)}^{4}\\ -\pi^{2}K_{11}{\left(\frac{m}{a}\right)}^{2}-\pi^{2}K_{12}{\left(\frac{n}{b}\right)}^{2}+K_{13} \end{array}\right]{\left(-1\right)}^{\frac{m+n}{2}-1}}{mn\left[\begin{array}{l} -\pi^{2}K_{1}{\left(\frac{m}{a}\right)}^{6}-\pi^{2}K_{2}{\left(\frac{m}{a}\right)}^{4}{\left(\frac{n}{b}\right)}^{2}-\pi^{2}K_{3}{\left(\frac{m}{a}\right)}^{2}{\left(\frac{n}{b}\right)}^{4}\\ -\pi^{2}K_{4}{\left(\frac{n}{b}\right)}^{6}+K_{5}{\left(\frac{m}{a}\right)}^{4}+K_{6}{\left(\frac{m}{a}\right)}^{2}{\left(\frac{n}{b}\right)}^{2}+K_{7}{\left(\frac{n}{b}\right)}^{4} \end{array}\right]}$$

where

(3) $$K_{1}=\frac{D_{xy}D_{x}}{2S_{y}};\;K_{2}=\frac{D_{xy}D_{x}}{2S_{x}}+\frac{D_{x}D_{y}-D_{xy}D_{x}\nu_{y}^{b}}{S_{y}};\;\;\;K_{3}=\frac{D_{xy}D_{y}}{2S_{y}}+\frac{D_{x}D_{y}-D_{xy}D_{x}\nu_{y}^{b}}{S_{x}};\;K_{4}=\frac{D_{xy}D_{y}}{2S_{x}};\;K_{5}=-D_{x};\;\;K_{6}=-2\left[D_{xy}\left(1-\nu_{x}^{b}\nu_{y}^{b}\right)+D_{x}\nu_{y}^{b}\right];\;K_{7}=-D_{y}\;;\;K_{8}=-\frac{D_{xy}D_{x}}{2S_{x}S_{y}};\;\;\;K_{9}=-\frac{D_{x}D_{y}-D_{xy}D_{x}\nu_{y}^{b}}{S_{x}S_{y}};\;K_{10}=-\frac{D_{xy}D_{y}}{2S_{x}S_{y}}\;K_{11}=\frac{D_{xy}\left(1-\nu_{x}^{b}\nu_{y}^{b}\right)}{2S_{y}}+\frac{D_{x}}{S_{x}};\;K_{12}=\frac{D_{xy}\left(1-\nu_{x}^{b}\nu_{y}^{b}\right)}{2S_{x}}+\frac{D_{y}}{S_{y}};\;\;\;K_{13}=-\left(1-\nu_{x}^{b}\nu_{y}^{b}\right)$$

The complete analysis description is presented in Appendix A.

### 5.3. Large Deflection Coefficient B

As presented above, the coefficient *B* describes the plate’s large deflection response, with the plate being considered as a thin membrane. Normal membranes cannot have a movable (M) BC, so it is complicated to find previous publications displaying an expression for the membrane deflection. Wang and El-Sheikh [6] (p. 816) have analyzed an isotropic rectangular plate and have presented an approximated expression for *q* = *Aw* + *Bw*³ for M BCs:

(4) $$q=\frac{\pi^{6}}{64}\left[4Dw{\left(\frac{1}{a^{2}}+\frac{1}{b^{2}}\right)}^{2}+\frac{Ehw^{3}}{4}\left(\frac{1}{a^{4}}+\frac{1}{b^{4}}\right)\right]$$

Modifying this expression to an orthotropic plate and using only the *B* term suggests the expression:

(5) $$B=k\cdot h\cdot \left(\frac{{E_{x}}^{t}}{a^{4}}+\frac{{E_{y}}^{t}}{b^{4}}\right)$$

where the coefficient *k* includes all the numerical factors. 

**Figure 3:**
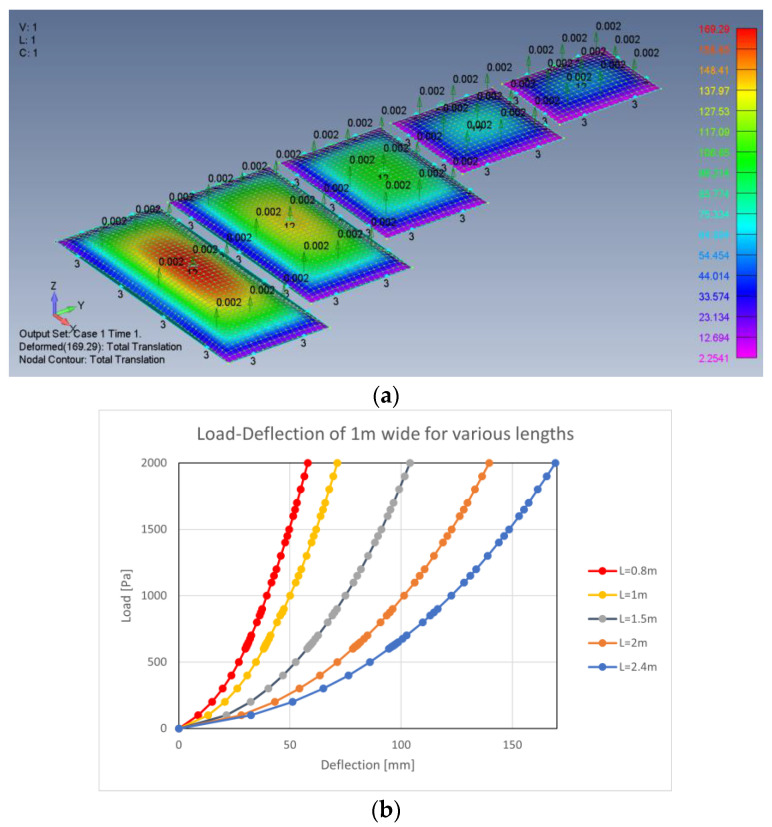
FEA deformation map (**a**) and load–deflection curves (**b**).

Since (5) is based only on the first term of a multiple term series, the result is not accurate enough to correctly represent the plates response. Therefore, finite element analyses (FEA) of 80 plates with various lengths and widths were performed. The FEA software was Femap with NX-Nastran version 2021.1 with its built-in non-linear static analysis. The FEA results are given in Appendix C, while the FEA information is given in Appendix D. Typical plate arrangements and mid-point load–deflection graphs are shown in Figure 3.

The values of *B* in (1) were calculated with a least-squares regression from the FEA results, and the following expression is suggested for *B*:

(6) $$B=h\sqrt{\frac{h}{a+b}}\left(\frac{E_{x}^{t}+E_{y}^{t}}{{\left(a+b\right)}^{4}}\right)K{\left(\frac{a}{b}\right)}^{p}$$

Note that the sum of the moduli and the sum of the length–width represent its averaged values, as the 2 division is included in *K*. The (*a*/*b*) is the aspect ratio of the plate. The *K* and *p* unitless values are *K* = 201.44 and *p* = −0.17165, while the units of the variables are: *B*: [Pa/m^3^], *h*: [m], *a*, *b*: [m], and *E*: [Pa]. Equation (6) describes multiwall plates well, but it is not necessarily suitable for other types of orthotropic plates.

## 6. Vacuum Chamber Test

**Figure 4:**
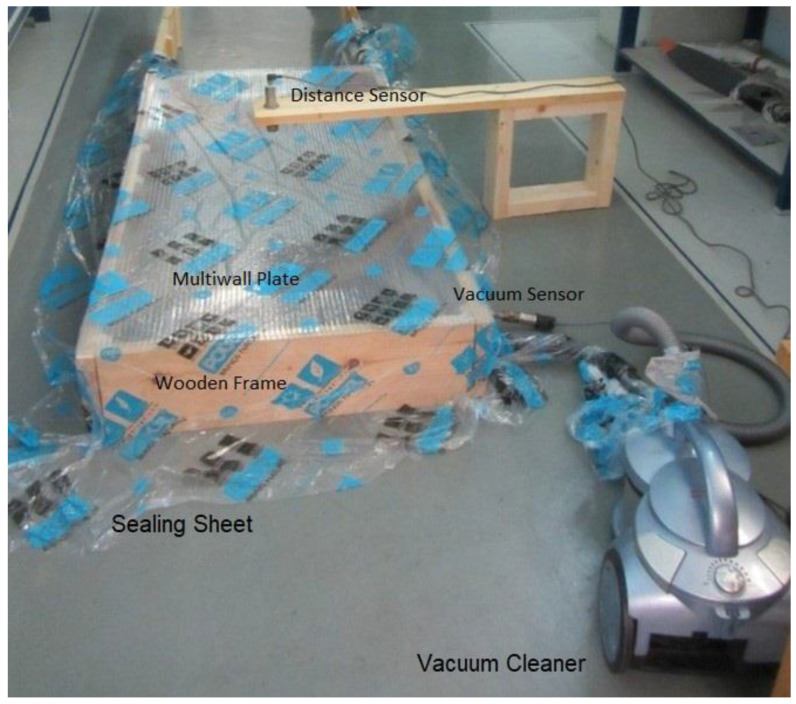
The vacuum chamber test set.

To check the multiwall plate response to transverse distributed load, a vacuum chamber test was used, as presented in Figure 4:

A 35 mm-thick wooden frame encloses a rectangular space with the required dimensions. The multiwall plate is freely placed on the frame’s edges. A thin plastic sheet covers the entire device and the floor near-by. A variable-speed vacuum cleaner is connected to the internal space through a drilled hole. The vacuum created causes the plastic sheet to seal all air leakages, allowing the vacuum level to gradually increase.

An electronic vacuum sensor measures the vacuum level through another hole in the wooden frame. The sensor is connected to a controller, which displays the data. Additionally, an ultrasonic distance sensor is placed 20 cm above the plate mid-point, measures the plate deflection, and transmits it to the controller. The vacuum units are [Pa], and the distance units are [mm].

The controller has a zero button to zero the vacuum and the distance values as the test starts. During the test, the vacuum level is gradually increased, while both load and deflection are recorded. Various local buckling phenomena are also closely monitored and recorded.

The plate edges are free to move on the wood frame, creating the M movable BC. As the vacuum level increases during the test, the thin plastic sealing sheet experiences tension and presses the plate edge to the wood frame. This may change the BC from SSSS to be somewhat closer to CCCC.

Several tests were performed at the Krumbein Structures Laboratory, Faculty of Aerospace Engineering—Technion. Next, a typical test is presented.

## 7. Results

There were two plates tested: length × width 1.5 × 0.8 m and length × width 0.8 × 1.5 m. The opening dimensions were 0.07 m less, i.e., 1.43 × 0.73 m.

## 8. Plate Details

- Type: 10 mm-PC double wall
- Nominal Area Weight 1700 g/m^2^

The measured results were least-squares fitted to the expression *q* = *Aw* + *Bw*³ and were compared to the theoretical curves.

**Figure 5:**
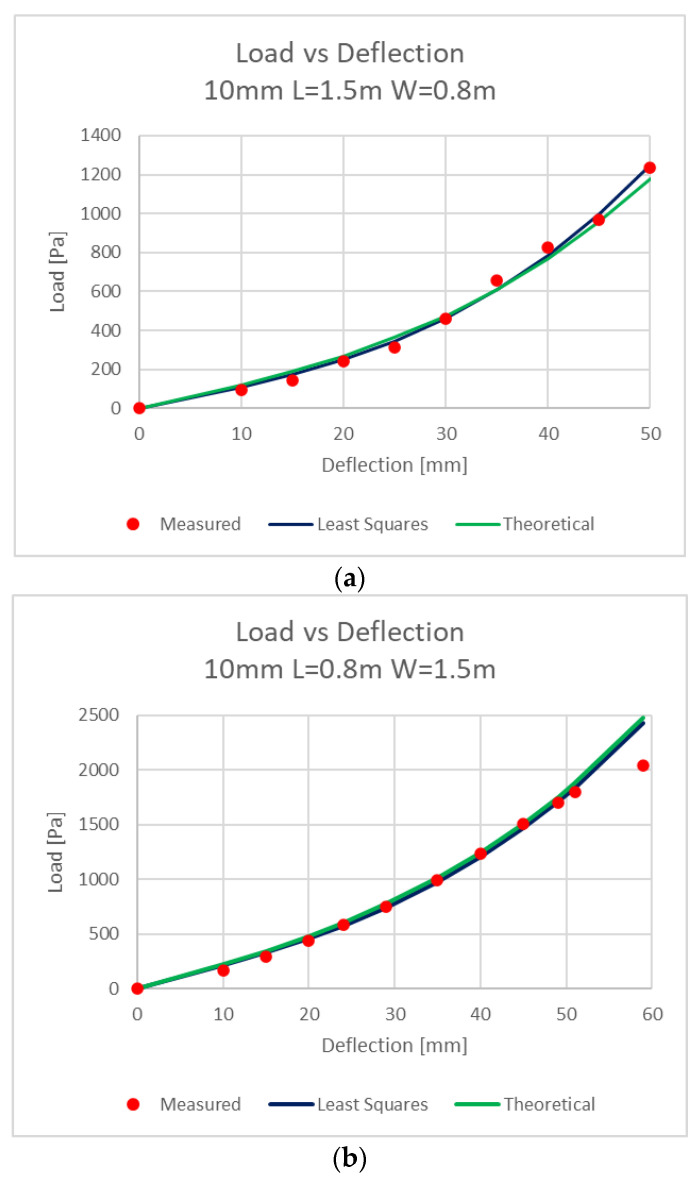
Load–Deflection graphs: (**a**) length 1.5 m, width 0.8 m; (**b**) length 0.8 m, width 1.5 m.

Part of the tested plates data are shown in the following Figure 5, Table 1 and Table 2:

The coefficients *A* and *B* were theoretically calculated with the expressions (2), (6), and they were compared to the measured values. The comparison is shown in Table 2:

As displayed in Table 2, the calculated coefficients comply to the measured one with some differences. 

## 9. FEA Results

**Figure 6:**
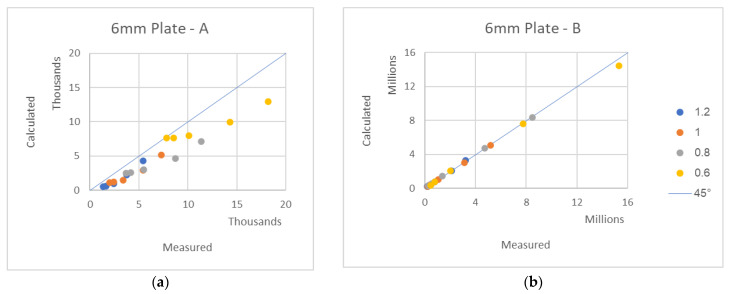

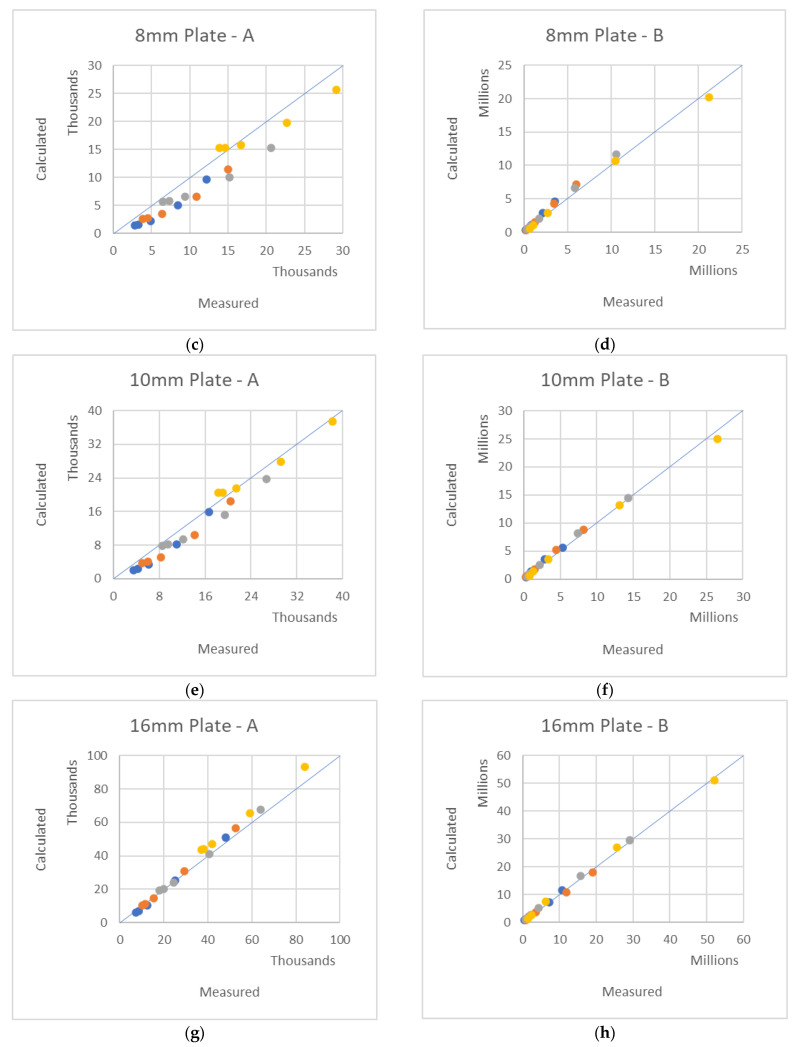
FEA-measured-calculated *A*, *B* expressions—a comparison of various plates: (**a**) 6 mm A, (**b**) 6 mm B with legend, (**c**) 8 mm A, (**d**) 8 mm B, (**e**) 10 mm A, (**f**) 10 mm B, (**g**) 16 mm A, (**h**) 16 mm B.

To find the response of the plates to transversal uniform load, 80 finite element analyses were performed: 4 plate types with 4 various widths and 5 different lengths (see in Figure 3 one of the tests). The coefficients *A* and *B* for each plate were both theoretically calculated using Equations (2) and (6), respectively, and they were also found from the graphs drawn using Equation (1). A comparison of the theoretically calculated *A* and *B* coefficients and the FEA-measured coefficients is presented in Figure 6. The legend at (b) applies to all other graphs.

The 45° lines represent the location of the perfect agreement between the theory and measurements. As it is shown in Figure 6, a very good agreement between the theory and analysis is found.

## 10. Large Number of Loading Tests

One of the manufacturers of PC multiwall plates is Plazit-Polygal (Plaskolite). During the years 2001–2002, they performed a large number of vacuum loading tests similar to the one described here. Plazit-Polygal has allowed the authors to use the test data for research and publication purposes. This permission is very much appreciated.

From about 250 tests, 120 tests of plates 6–16 mm thick were chosen. Each test has a set of measured load–deflection values for various width–length measurements. The analysis of every test was a linear least-squares regression that calculated the coefficients *A* and *B* in the expression *q* = *Aw* + *Bw*³. The results are listed in Appendix C. The coefficient of determination *R*² (goodness-of-fit) in all tested cases was above 0.99. These very good fits support the validity of the suggested *A, B* large deflection approximation (Equation (1)).

**Figure 7:**
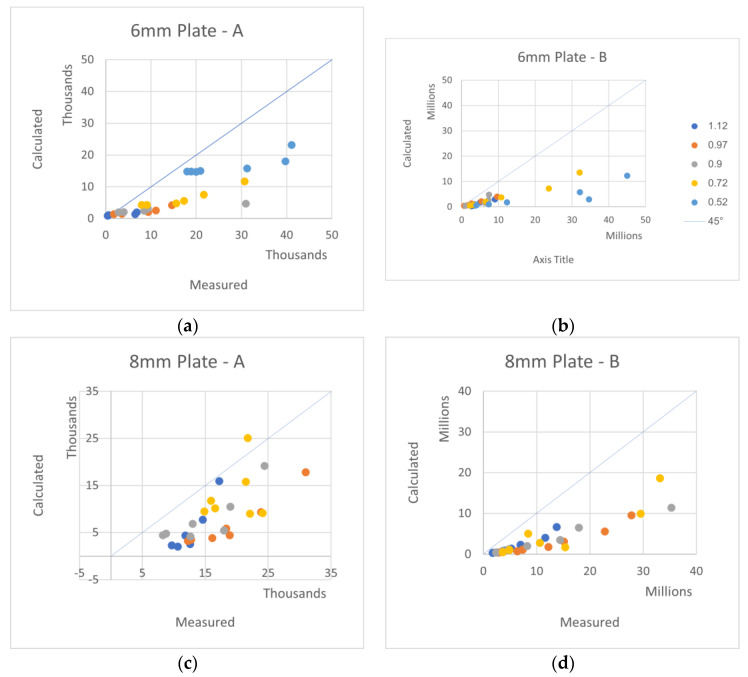

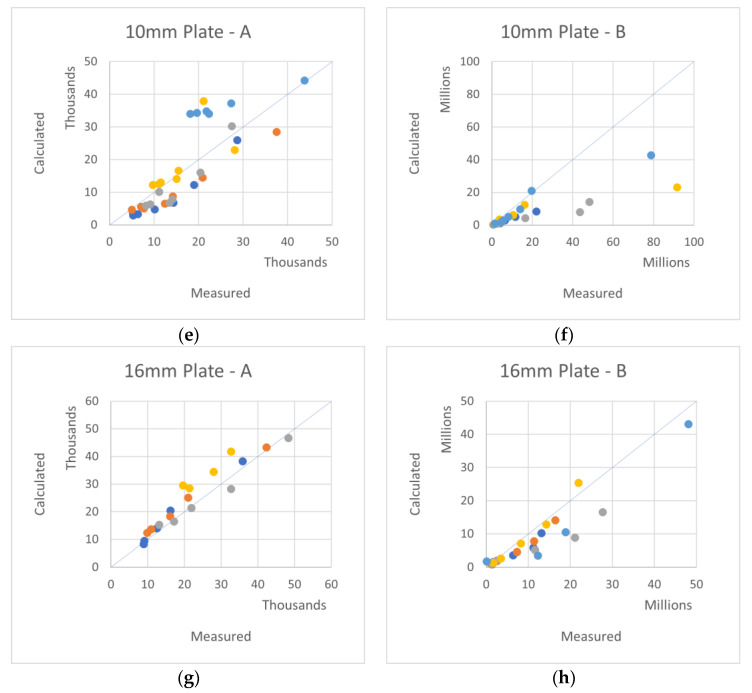
Measured–Calculated *A*, *B* constants—a comparison of various plates. (**a**) 6 mm A, (**b**) 6 mm B with legend, (**c**) 8 mm A, (**d**) 8 mm B, (**e**) 10 mm A, (**f**) 10 mm B, (**g**) 16 mm A, (**h**) 16 mm B.

**Figure 8:**
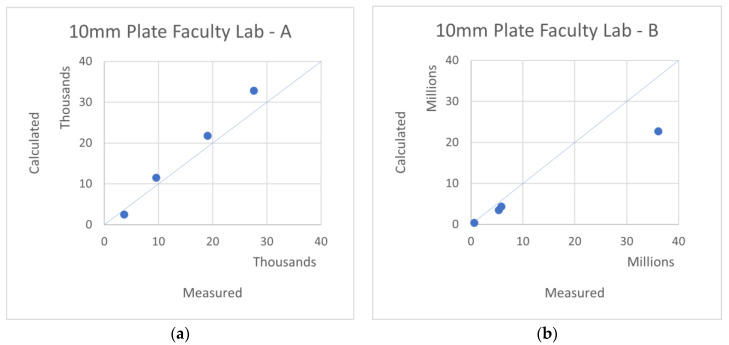
Measured–Calculated *A*, *B*—a comparison of various plates performed recently at the Aerospace Lab. (**a**) 10 mm A, (**b**) 10 mm B.

The equivalent elastic constants and moduli of the plates were measured and given with the test data in Appendix B. This information allows for the calculation of the *A, B* coefficients according to the theory above. The measured and calculated *A, B* coefficients are compared in Figure 7 and Figure 8, where the unit of *A* is [Pa/m] and the unit of *B* is [Pa/m^3^].

In Figure 7 and Figure 8, the horizontal axes are the *A* and *B* measured coefficients, while the vertical axes are the theoretically calculated coefficients. The 45° lines represent the location of the perfect agreement between the theory and measurements. The various colors are for various plate widths [m], as shown in the Figure 6b graph legend.

Generally, the theory–measurement agreements are better for the coefficient *A* over the coefficient *B*, and they are also better for higher thickness over lower thickness. 

Since Polygal’s tests were performed more than 20 years ago, the tested samples are not available for verification anymore. Many dimensions of the plates were missing in the records, so the standard values were taken from the plate’s data sheets. Nevertheless, the actual real plate dimension values almost always deviate from the standard values, as may occur in real manufacturing. These deviations can be rather significant and, for sure, influence on the plate rigidities. This is probably the main source for the inconsistencies in the reported data.

As the vacuum was measured at that time with a water manometer, which is not accurate enough, it is very possible that errors do exist in the data. These errors can be seen in Appendix C, Table A4, Table A5, Table A6 and Table A7, where the measured coefficients *A* should monotonically increase while the length values decrease, but in several cases, they unexpectedly decrease.

By being very comprehensive, with a large number of length/width combinations, the understanding of the plate response to transverse pressure still has some value, besides proving the *q* = *Aw* + *Bw*³ response.

Note that the last plate test (10 mm Plate Faculty Lab) was performed more recently, under a better-controlled environment, and therefore, it presents a more accurate agreement.

## 11. Conclusions

Large deflection of multiwall plates, under distributed transverse pressure and SSSS-M boundary conditions, has been found to comply with the *q* = *Aw* + *Bw*³ approximation rule with very good *R*² (goodness-of-fit) values.

The suggested expressions for *A* and *B* coefficients have good agreement with FEA results, while in actual tests, they appear to be more applicable for thicker plates.

The deviations in the presented experimental loading data may relate to the measurement technique. Good laboratory practice would lead to more accurate results.

## Figures and Tables

**Table 1:** The elastic constants of this multiwall plate.

Thickness	*h*	[mm]	10
Area Weight	*W*	[g/m²]	1713
Walls Thickness	*t_w_*	[mm]	1.154
Equivalent *G*	*G_eq_*	[MPa]	100.348
For small deflection coefficient *A*:			
	*D_x_*	[Nm]	70.12106
Bending:	*D_y_*	[Nm]	54.10356
	*D_xy_*	[Nm]	8.362
Shear:	*S_x_*	[N/m]	59,890.03
	*S_y_*	[N/m]	1662.076
	*ν_x_^b^*		0.38
	*ν_y_^b^* = *D_y_*/*D_x_* ∗ *ν_x_^b^*		0.293
For large deflection coefficient *B*:			
	*E_x_^t^*	[MPa]	342.60
Tension:	*E_y_^t^*	[MPa]	276.96
	*ν_x_^t^*		0.38
	*ν_y_^t^* = *E_y_^t^*/*E_x_^t^* ∗ *ν_x_^t^*		0.307

**Table 2:** Coefficients results.

Coefficient	Length 1.5 m, Width 0.8 m			Length 0.8 m, Width 1.5 m		
	Measured	Theoretical	% Difference	Measured	Theoretical	% Difference
*A* [Pa/m]	9602	11,487 (2)	16.4%	19,061	21,805	12.6%
*B* [Pa/m³]	5,370,163	3,475,816 (6)	35.3%	5,858,384	4,378,294	25.3%

**Figure A1:**
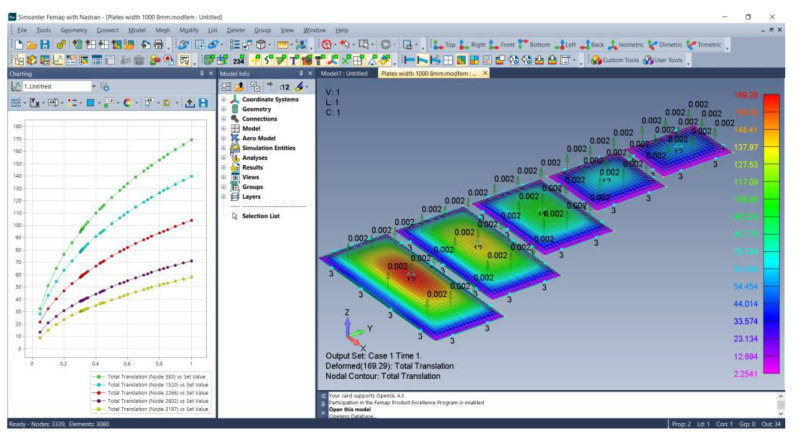
Femap typical screen.

## Data Availability

The data presented in this study are available in this article.

## Appendix A

### Appendix A.1. Small Deflections Coefficient A—Libove’s and Reddy’s Solutions

A multiwall plate has considerable transverse shear flexibility in the width direction *y*, while it is relatively rigid to shear in the length direction *x*. Nevertheless, the analysis here considers both directions, omitting the shear in the length direction only at the end of the derivation.

### Appendix A.2. Libove’s Solution

In the following, Poisson’s ratios are designated $\mu_{x}{,\mu }_{y}$ as they appear in the source, while in the main paper, they are designated $\nu_{x}^{b}$, $\nu_{y}^{b}$.

Libove’s NACA Report No. 899 [17] presents the PDEs describing this problem.

The three main equations are on page 144 of [17], between (12) and (13), without numbers. The notations are shown in [17] on page 140.

The following Equations (A1)–(A3) are simply copied from [17]:

(A1) $$\left(N_{x}\frac{\partial^{2}}{\partial x^{2}}+N_{y}\frac{\partial^{2}}{\partial y^{2}}+2N_{xy}\frac{\partial^{2}}{\partial x\partial y}\right)w+\left(\frac{\partial }{\partial x}\right)Q_{x}+\left(\frac{\partial }{\partial y}\right)Q_{y}=-q$$

(A2) $$\begin{array}{c} \left[-D_{xy}\frac{\partial^{3}}{\partial x\partial y^{2}}-\frac{D_{x}}{1-\mu_{x}\mu_{y}}\left(\mu_{y}\frac{\partial^{3}}{\partial x\partial y^{2}}+\frac{\partial^{3}}{\partial x^{3}}\right)\right]w+\left[\frac{1}{2}\frac{D_{xy}}{D_{Qx}}\frac{\partial^{2}}{\partial y^{2}}+\frac{D_{x}}{\left(1-\mu_{x}\mu_{y}\right)D_{Qx}}\frac{\partial^{2}}{\partial x^{2}}-1\right]Q_{x}+\\ \left[\frac{1}{2}\frac{D_{xy}}{D_{Qy}}\frac{\partial^{2}}{\partial x\partial y}+\frac{D_{x}\mu_{y}}{\left(1-\mu_{x}\mu_{y}\right)D_{Qy}}\frac{\partial^{2}}{\partial x\partial y}\right]Q_{y}=0 \end{array}$$

(A3) $$\begin{array}{c} \left[-D_{xy}\frac{\partial^{3}}{\partial x^{2}\partial y}-\frac{D_{y}}{1-\mu_{x}\mu_{y}}\left(\mu_{x}\frac{\partial^{3}}{\partial x^{2}\partial y}+\frac{\partial^{3}}{\partial y^{3}}\right)\right]w+\left[\frac{1}{2}\frac{D_{xy}}{D_{Qx}}\frac{\partial^{2}}{\partial x\partial y}+\frac{D_{y}\mu_{x}}{\left(1-\mu_{x}\mu_{y}\right)D_{Qx}}\frac{\partial^{2}}{\partial x\partial y}\right]Q_{x}+\\ \left[\frac{1}{2}\frac{D_{xy}}{D_{Qy}}\frac{\partial^{2}}{\partial x^{2}}+\frac{D_{y}}{\left(1-\mu_{x}\mu_{y}\right)D_{Qy}}\frac{\partial^{2}}{\partial y^{2}}-1\right]Q_{y}=0 \end{array}$$

In order to have it in a more readable form, we change the shear rigidity $D_{Qx}$ to be $S_{x}$ and the $D_{Qy}$ to be $S_{y}$. After opening the parenthesis, we get:

(A4) $$N_{x}\frac{\partial^{2}w}{\partial x^{2}}+N_{y}\frac{\partial^{2}w}{\partial y^{2}}+2N_{xy}\frac{\partial^{2}w}{\partial x\partial y}+\frac{\partial Q_{x}}{\partial x}+\frac{\partial Q_{y}}{\partial y}=-q$$

(A5) $$\begin{array}{c} -\left[D_{xy}+\frac{\mu_{y}D_{x}}{\left(1-\mu_{x}\mu_{y}\right)}\right]\frac{\partial^{3}w}{\partial x\partial y^{2}}-\frac{D_{x}}{\left(1-\mu_{x}\mu_{y}\right)}\frac{\partial^{3}w}{\partial x^{3}}+\frac{D_{xy}}{2S_{x}}\frac{\partial^{2}Q_{x}}{\partial y^{2}}+\frac{D_{x}}{\left(1-\mu_{x}\mu_{y}\right)S_{x}}\frac{\partial^{2}Q_{x}}{\partial x^{2}}-Q_{x}+\\ \left[\frac{D_{xy}}{2S_{y}}+\frac{\mu_{y}D_{x}}{\left(1-\mu_{x}\mu_{y}\right)S_{y}}\right]\frac{\partial^{2}Q_{y}}{\partial x\partial y}=0 \end{array}$$

(A6) $$\begin{array}{c} -\left[D_{xy}+\frac{\mu_{x}D_{y}}{\left(1-\mu_{x}\mu_{y}\right)}\right]\frac{\partial^{3}w}{\partial x^{2}\partial y}-\frac{D_{y}}{\left(1-\mu_{x}\mu_{y}\right)}\frac{\partial^{3}w}{\partial y^{3}}+\left[\frac{D_{xy}}{2S_{x}}+\frac{\mu_{x}D_{y}}{\left(1-\mu_{x}\mu_{y}\right)S_{x}}\right]\frac{\partial^{2}Q_{x}}{\partial x\partial y}+\frac{D_{xy}}{2S_{y}}\frac{\partial^{2}Q_{y}}{\partial x^{2}}+\\ \frac{D_{y}}{\left(1-\mu_{x}\mu_{y}\right)S_{y}}\frac{\partial^{2}Q_{y}}{\partial y^{2}}-Q_{y}=0 \end{array}$$

Some simplification can be done by taking $N_{x}$, $N_{y}$, and $N_{xy}$ to be zero because no membrane forces exist in our problem.

After doing so and repeating Equations (A5) and (A6) in Equations (A8) and (A9), we get:

(A7) $$\frac{\partial Q_{x}}{\partial x}+\frac{\partial Q_{y}}{\partial y}=-q$$

(A8) $$\begin{array}{c} -\left[D_{xy}+\frac{\mu_{y}D_{x}}{\left(1-\mu_{x}\mu_{y}\right)}\right]\frac{\partial^{3}w}{\partial x\partial y^{2}}-\frac{D_{x}}{\left(1-\mu_{x}\mu_{y}\right)}\frac{\partial^{3}w}{\partial x^{3}}+\frac{D_{xy}}{2S_{x}}\frac{\partial^{2}Q_{x}}{\partial y^{2}}+\frac{D_{x}}{\left(1-\mu_{x}\mu_{y}\right)S_{x}}\frac{\partial^{2}Q_{x}}{\partial x^{2}}+\\ \left[\frac{D_{xy}}{2S_{y}}+\frac{\mu_{y}D_{x}}{\left(1-\mu_{x}\mu_{y}\right)S_{y}}\right]\frac{\partial^{2}Q_{y}}{\partial x\partial y}=Q_{x} \end{array}$$

(A9) $$\begin{array}{c} -\left[D_{xy}+\frac{\mu_{x}D_{y}}{\left(1-\mu_{x}\mu_{y}\right)}\right]\frac{\partial^{3}w}{\partial x^{2}\partial y}-\frac{D_{y}}{\left(1-\mu_{x}\mu_{y}\right)}\frac{\partial^{3}w}{\partial y^{3}}+\left[\frac{D_{xy}}{2S_{x}}+\frac{\mu_{x}D_{y}}{\left(1-\mu_{x}\mu_{y}\right)S_{x}}\right]\frac{\partial^{2}Q_{x}}{\partial x\partial y}+\frac{D_{xy}}{2S_{y}}\frac{\partial^{2}Q_{y}}{\partial x^{2}}+\\ \frac{D_{y}}{\left(1-\mu_{x}\mu_{y}\right)S_{y}}\frac{\partial^{2}Q_{y}}{\partial y^{2}}=Q_{y} \end{array}$$

Equations (A7)–(A9) are a linear set of three PDEs. It should be solved simultaneously for the unknowns $Q_{x}$, $Q_{y}$, and w for the independent load *q*. All the other coefficients are already known before.

According to [17], this set can be separated to be three dual variable PDEs for every unknown in terms of the load *q*. One of them is for *w*, which is a sixth order equation, as shown in [17] (13a):

(A10) $$\left[D\right]w=-\left[M\right]q$$

where the operators [*D*] and [*M*], after the omission of *N* terms, are:

(A11) $$\begin{array}{c} \left[D\right]=\frac{D_{xy}D_{x}}{2S_{y}}\frac{\partial^{6}}{\partial x^{6}}+\left(\frac{D_{xy}D_{x}}{2S_{x}}+\frac{D_{x}D_{y}-\frac{1}{2}D_{xy}D_{x}\mu_{y}-\frac{1}{2}D_{xy}D_{y}\mu_{x}}{S_{y}}\right)\frac{\partial^{6}}{\partial x^{4}\partial y^{2}}+\\ \left(\frac{D_{xy}D_{y}}{2S_{y}}+\frac{D_{x}D_{y}-\frac{1}{2}D_{xy}D_{x}\mu_{y}-\frac{1}{2}D_{xy}D_{y}\mu_{x}}{S_{x}}\right)\frac{\partial^{6}}{\partial x^{2}\partial y^{4}}+\frac{D_{xy}D_{y}}{2S_{x}}\frac{\partial^{6}}{\partial y^{6}}-D_{x}\frac{\partial^{4}}{\partial x^{4}}-\\ \left[2D_{xy}\left(1-\mu_{x}\mu_{y}\right)+D_{x}\mu_{y}+D_{y}\mu_{x}\right]\frac{\partial^{4}}{\partial x^{2}\partial y^{2}}-D_{y}\frac{\partial^{4}}{\partial y^{4}} \end{array}$$

(A12) $$\begin{array}{c} \left[M\right]=\frac{D_{xy}D_{x}}{2{S_{x}S}_{y}}\frac{\partial^{4}}{\partial x^{4}}+\frac{D_{x}D_{y}-\frac{1}{2}D_{xy}D_{x}\mu_{y}-\frac{1}{2}D_{xy}D_{y}\mu_{x}}{S_{x}S_{y}}\frac{\partial^{4}}{\partial x^{2}\partial y^{2}}+\frac{D_{xy}D_{y}}{2S_{x}S_{y}}\frac{\partial^{4}}{\partial y^{4}}-\\ \left(\frac{D_{xy}\left(1-\mu_{x}\mu_{y}\right)}{2S_{x}}+\frac{D_{y}}{S_{y}}\right)\frac{\partial^{2}}{\partial y^{2}}-\left(\frac{D_{xy}\left(1-\mu_{x}\mu_{y}\right)}{2S_{y}}+\frac{D_{x}}{S_{x}}\right)\frac{\partial^{2}}{\partial x^{2}}+\left(1-\mu_{x}\mu_{y}\right) \end{array}$$

After replacing $D_{y}\mu_{x}$ with $D_{x}\mu_{y}$ in the above, according to [17] (8), Equation (A10) becomes:

(A13) $$\begin{array}{c} \frac{D_{xy}D_{x}}{2S_{y}}\frac{\partial^{6}w}{\partial x^{6}}+\left(\frac{D_{xy}D_{x}}{2S_{x}}+\frac{D_{x}D_{y}-D_{xy}D_{x}\mu_{y}}{S_{y}}\right)\frac{\partial^{6}w}{\partial x^{4}\partial y^{2}}+\left(\frac{D_{xy}D_{y}}{2S_{y}}+\frac{D_{x}D_{y}-D_{xy}D_{x}\mu_{y}}{S_{x}}\right)\frac{\partial^{6}w}{\partial x^{2}\partial y^{4}}+\\ \frac{D_{xy}D_{y}}{2S_{x}}\frac{\partial^{6}w}{\partial y^{6}}-D_{x}\frac{\partial^{4}w}{\partial x^{4}}-2\left[D_{xy}\left(1-\mu_{x}\mu_{y}\right)+D_{x}\mu_{y}\right]\frac{\partial^{4}w}{\partial x^{2}\partial y^{2}}-D_{y}\frac{\partial^{4}w}{\partial y^{4}}=\\ -\frac{D_{xy}D_{x}}{2S_{x}S_{y}}\frac{\partial^{4}q}{\partial x^{4}}-\frac{D_{x}D_{y}-D_{xy}D_{x}\mu_{y}}{S_{x}S_{y}}\frac{\partial^{4}q}{\partial x^{2}\partial y^{2}}-\frac{D_{xy}D_{y}}{2S_{x}S_{y}}\frac{\partial^{4}q}{\partial y^{4}}+\left(\frac{D_{xy}\left(1-\mu_{x}\mu_{y}\right)}{2S_{x}}+\frac{D_{y}}{S_{y}}\right)\frac{\partial^{2}q}{\partial y^{2}}+\\ +\left(\frac{D_{xy}\left(1-\mu_{x}\mu_{y}\right)}{2S_{y}}+\frac{D_{x}}{S_{x}}\right)\frac{\partial^{2}q}{\partial x^{2}}-\left(1-\mu_{x}\mu_{y}\right)q \end{array}$$

or in a shorter form:

(A14) $$\begin{array}{c} K_{1}\frac{\partial^{6}w}{\partial x^{6}}+K_{2}\frac{\partial^{6}w}{\partial x^{4}\partial y^{2}}+K_{3}\frac{\partial^{6}w}{\partial x^{2}\partial y^{4}}+K_{4}\frac{\partial^{6}w}{\partial y^{6}}+K_{5}\frac{\partial^{4}w}{\partial x^{4}}+K_{6}\frac{\partial^{4}w}{\partial x^{2}\partial y^{2}}+K_{7}\frac{\partial^{4}w}{\partial y^{4}}=\\ K_{8}\frac{\partial^{4}q}{\partial x^{4}}+K_{9}\frac{\partial^{4}q}{\partial x^{2}\partial y^{2}}+K_{10}\frac{\partial^{4}q}{\partial y^{4}}+K_{11}\frac{\partial^{2}q}{\partial x^{2}}+K_{12}\frac{\partial^{2}q}{\partial y^{2}}+K_{13}q \end{array}$$

where the coefficients *K_i_* are:

(A15) $$K_{1}=\frac{D_{xy}D_{x}}{2S_{y}};\;K_{2}=\frac{D_{xy}D_{x}}{2S_{x}}+\frac{D_{x}D_{y}-D_{xy}D_{x}\mu_{y}}{S_{y}};\;K_{3}=\frac{D_{xy}D_{y}}{2S_{y}}+\frac{D_{x}D_{y}-D_{xy}D_{x}\mu_{y}}{S_{x}}\;K_{4}=\frac{D_{xy}D_{y}}{2S_{x}};\;K_{5}=-D_{x};\;K_{6}=-2\left[D_{xy}\left(1-\mu_{x}\mu_{y}\right)+D_{x}\mu_{y}\right];\;K_{7}=-D_{y}\;K_{8}=-\frac{D_{xy}D_{x}}{2S_{x}S_{y}};\;K_{9}=-\frac{D_{x}D_{y}-D_{xy}D_{x}\mu_{y}}{S_{x}S_{y}};\;K_{10}=-\frac{D_{xy}D_{y}}{2S_{x}S_{y}}\;\begin{array}{c} K_{11}=\frac{D_{xy}\left(1-\mu_{x}\mu_{y}\right)}{2S_{y}}+\frac{D_{x}}{S_{x}}\;;\;K_{12}=\frac{D_{xy}\left(1-\mu_{x}\mu_{y}\right)}{2S_{x}}+\frac{D_{y}}{S_{y}}\;;\;K_{13}=-\left(1-\mu_{x}\mu_{y}\right) \end{array}$$

The following is a standard Fourier series solution of a linear PDE.

The deflection boundary conditions are all *w* = 0, so we can assume a double sine series as the solution (Navier Solution):

(A16) $$w\left(x,y\right)=\sum_{n=1}^{\infty }\sum_{m=1}^{\infty }W_{mn}\sin\frac{m\pi x}{a}\sin\frac{n\pi y}{b}$$

where $W_{mn}$ are coefficients to be determined. Substituting (A16) into (A14) yields:

(A17) $$\begin{array}{c} \sum_{n=1}^{\infty }\sum_{m=1}^{\infty }\left[\begin{array}{l} -K_{1}{\left(\frac{m\pi }{a}\right)}^{6}-K_{2}{\left(\frac{m\pi }{a}\right)}^{4}{\left(\frac{n\pi }{b}\right)}^{2}-K_{3}{\left(\frac{m\pi }{a}\right)}^{2}{\left(\frac{n\pi }{b}\right)}^{4}\\ -K_{4}{\left(\frac{n\pi }{b}\right)}^{6}+K_{5}{\left(\frac{m\pi }{a}\right)}^{4}+K_{6}{\left(\frac{m\pi }{a}\right)}^{2}{\left(\frac{n\pi }{b}\right)}^{2}+K_{7}{\left(\frac{n\pi }{b}\right)}^{4} \end{array}\right]W_{mn}\sin\frac{m\pi x}{a}\sin\frac{n\pi y}{b}=\\ K_{8}\frac{\partial^{4}q}{\partial x^{4}}+K_{9}\frac{\partial^{4}q}{\partial x^{2}\partial y^{2}}+K_{10}\frac{\partial^{4}q}{\partial y^{4}}+K_{11}\frac{\partial^{2}q}{\partial x^{2}}+K_{12}\frac{\partial^{2}q}{\partial y^{2}}+K_{13}q \end{array}$$

This suggests that the equation’s right-hand side should also be expanded into a double sine series:

(A18) $$q\left(x,y\right)=\sum_{n=1}^{\infty }\sum_{m=1}^{\infty }q_{mn}\sin\frac{m\pi x}{a}\sin\frac{n\pi y}{b}$$

So (A17) becomes:

(A19) $$\sum_{n=1}^{\infty }\sum_{m=1}^{\infty }\left\{\begin{array}{l} \left[\begin{array}{l} -K_{1}{\left(\frac{m\pi }{a}\right)}^{6}-K_{2}{\left(\frac{m\pi }{a}\right)}^{4}{\left(\frac{n\pi }{b}\right)}^{2}-K_{3}{\left(\frac{m\pi }{a}\right)}^{2}{\left(\frac{n\pi }{b}\right)}^{4}\\ -K_{4}{\left(\frac{n\pi }{b}\right)}^{6}+K_{5}{\left(\frac{m\pi }{a}\right)}^{4}+K_{6}{\left(\frac{m\pi }{a}\right)}^{2}{\left(\frac{n\pi }{b}\right)}^{2}+K_{7}{\left(\frac{n\pi }{b}\right)}^{4} \end{array}\right]W_{mn}\\ -\left[\begin{array}{l} K_{8}{\left(\frac{m\pi }{a}\right)}^{4}+K_{9}{\left(\frac{m\pi }{a}\right)}^{2}{\left(\frac{n\pi }{b}\right)}^{2}+K_{10}{\left(\frac{n\pi }{b}\right)}^{4}\\ -K_{11}{\left(\frac{m\pi }{a}\right)}^{2}-K_{12}{\left(\frac{n\pi }{b}\right)}^{2}+K_{13} \end{array}\right]q_{mn} \end{array}\right\}\sin\frac{m\pi x}{a}\sin\frac{n\pi y}{b}=0$$

Since (A19) must exist at all points *x*, *y* in the domain, so the coefficients of $\sin\frac{m\pi x}{a}\sin\frac{n\pi y}{b}$ must be zero for every *m* and *n*. This yields:

(A20) $$W_{mn}=\frac{\left[\begin{array}{l} K_{8}{\left(\frac{m\pi }{a}\right)}^{4}+K_{9}{\left(\frac{m\pi }{a}\right)}^{2}{\left(\frac{n\pi }{b}\right)}^{2}+K_{10}{\left(\frac{n\pi }{b}\right)}^{4}\\ -K_{11}{\left(\frac{m\pi }{a}\right)}^{2}-K_{12}{\left(\frac{n\pi }{b}\right)}^{2}+K_{13} \end{array}\right]q_{mn}}{\left[\begin{array}{l} -K_{1}{\left(\frac{m\pi }{a}\right)}^{6}-K_{2}{\left(\frac{m\pi }{a}\right)}^{4}{\left(\frac{n\pi }{b}\right)}^{2}-K_{3}{\left(\frac{m\pi }{a}\right)}^{2}{\left(\frac{n\pi }{b}\right)}^{4}\\ -K_{4}{\left(\frac{n\pi }{b}\right)}^{6}+K_{5}{\left(\frac{m\pi }{a}\right)}^{4}+K_{6}{\left(\frac{m\pi }{a}\right)}^{2}{\left(\frac{n\pi }{b}\right)}^{2}+K_{7}{\left(\frac{n\pi }{b}\right)}^{4} \end{array}\right]}$$

For an evenly distributed load *q*(*x,y*) *= q*_o_, the coefficients *q_mn_* are:

(A21) $$q_{mn}=\frac{16q_{0}}{\pi^{2}mn}\;m,n=1,3,5,7\ldots$$

We are interested in the deflection at the middle point of the plate $w_{max}$, where $x=\frac{a}{2};\;y=\frac{b}{2}$, and $\sin\frac{m\pi x}{a}\sin\frac{n\pi y}{b}={\left(-1\right)}^{\frac{m+n}{2}-1}$.

The final solution is therefore:

(A22) $$w_{max}=\frac{16q_{0}}{\pi^{6}}\sum_{n=1,3,5\ldots }^{\infty }\sum_{m=1,3,5\ldots }^{\infty }\frac{\left[\begin{array}{l} \pi^{4}K_{8}{\left(\frac{m}{a}\right)}^{4}+\pi^{4}K_{9}{\left(\frac{m}{a}\right)}^{2}{\left(\frac{n}{b}\right)}^{2}+\pi^{4}K_{10}{\left(\frac{n}{b}\right)}^{4}\\ -\pi^{2}K_{11}{\left(\frac{m}{a}\right)}^{2}-\pi^{2}K_{12}{\left(\frac{n}{b}\right)}^{2}+K_{13} \end{array}\right]{\left(-1\right)}^{\frac{m+n}{2}-1}}{mn\left[\begin{array}{l} -\pi^{2}K_{1}{\left(\frac{m}{a}\right)}^{6}-\pi^{2}K_{2}{\left(\frac{m}{a}\right)}^{4}{\left(\frac{n}{b}\right)}^{2}-\pi^{2}K_{3}{\left(\frac{m}{a}\right)}^{2}{\left(\frac{n}{b}\right)}^{4}\\ -\pi^{2}K_{4}{\left(\frac{n}{b}\right)}^{6}+K_{5}{\left(\frac{m}{a}\right)}^{4}+K_{6}{\left(\frac{m}{a}\right)}^{2}{\left(\frac{n}{b}\right)}^{2}+K_{7}{\left(\frac{n}{b}\right)}^{4} \end{array}\right]}$$

where the coefficients *K_i_* are given in (A15).

Equation (A22) presents the linear relationship between the load and the deflection in a small defection case:

(A23) $$w_{max}=A\cdot q_{0}$$

### Appendix A.3. Reddy’s Solution

J.N. Reddy [18] (p. 368), has also solved this problem. The numerical results of Reddy [18] are identical to those of Libove’s NACA 899 [17].

Nevertheless, to complete this comparison, the details of Reddy’s solution are repeated here with their original notations.

The elastic constant definitions used by Reddy are in Libove’s terms:

(A24) $$\begin{array}{c} D_{11}=\frac{D_{x}}{1-\mu_{x}\mu_{y}}\;;\;D_{12}=\frac{D_{x}\mu_{y}}{1-\mu_{x}\mu_{y}}\;;\;D_{22}=\frac{D_{y}}{1-\mu_{x}\mu_{y}}\;;\;D_{66}=\frac{1}{2}D_{xy};\;KA_{44}=S_{y}\;;\;KA_{55}=S_{x}\;;\\ \nu_{21}=\mu_{y}\;;\;\nu_{12}=\mu_{x} \end{array}$$

There are several easing assumptions that simplify the original Reddy’s expressions:

1. Deflections, strains, and rotations are small
2. No initial in-plane forces
3. Static state-no changes in time
4. No thermal loads
5. No elastic foundation

Under these assumptions, expressions [18] (10.1.31–35 at pp. 365–366) are:

(The original index 0, which represents middle plate value was omitted).

(A25) $$A_{11}\left(\frac{\partial^{2}u}{\partial x^{2}}+\frac{\partial w}{\partial x}\frac{\partial^{2}w}{\partial x^{2}}\right)+A_{12}\left(\frac{\partial^{2}v}{\partial y\partial x}+\frac{\partial w}{\partial y}\frac{\partial^{2}w}{\partial y\partial x}\right)+A_{66}\left(\frac{\partial^{2}u}{\partial y^{2}}+\frac{\partial^{2}v}{\partial x\partial y}+\frac{\partial^{2}w}{\partial x\partial y}\frac{\partial w}{\partial y}+\frac{\partial w}{\partial x}\frac{\partial^{2}w}{\partial y^{2}}\right)=0\;A_{66}\left(\frac{\partial^{2}u}{\partial y\partial x}+\frac{\partial^{2}v}{\partial x^{2}}+\frac{\partial^{2}w}{\partial x^{2}}\frac{\partial w}{\partial y}+\frac{\partial w}{\partial x}\frac{\partial^{2}w}{\partial y\partial x}\right)+A_{12}\left(\frac{\partial^{2}u}{\partial x\partial y}+\frac{\partial w}{\partial x}\frac{\partial^{2}w}{\partial x\partial y}\right)+A_{22}\left(\frac{\partial^{2}v}{\partial y^{2}}+\frac{\partial w}{\partial y}\frac{\partial^{2}w}{\partial y^{2}}\right)=0\;{K_{s}A}_{55}\left(\frac{\partial^{2}w}{\partial x^{2}}+\frac{\partial \phi_{x}}{\partial x}\right)+{K_{s}A}_{44}\left(\frac{\partial^{2}w}{\partial y^{2}}+\frac{\partial \phi_{y}}{\partial y}\right)+q\left(x,y\right)=0\;D_{11}\left(\frac{\partial^{2}\phi_{x}}{\partial x^{2}}\right)+D_{12}\left(\frac{\partial^{2}\phi_{y}}{\partial y\partial x}\right)+D_{66}\left(\frac{\partial^{2}\phi_{x}}{\partial y^{2}}+\frac{\partial^{2}\phi_{y}}{\partial y\partial x}\right)-{K_{s}A}_{55}\left(\frac{\partial w}{\partial x}+\phi_{x}\right)=0\;D_{66}\left(\frac{\partial^{2}\phi_{x}}{\partial x\partial y}+\frac{\partial^{2}\phi_{y}}{\partial x^{2}}\right)+D_{12}\left(\frac{\partial^{2}\phi_{x}}{\partial x\partial y}\right)+D_{22}\left(\frac{\partial^{2}\phi_{y}}{\partial y^{2}}\right)-{K_{s}A}_{44}\left(\frac{\partial w}{\partial y}+\phi_{y}\right)=0$$

The Navier solution with double Fourier series is:

(A26) $$q\left(x,y\right)=\sum_{n=1}^{\infty }\sum_{m=1}^{\infty }Q_{mn}\sin\frac{m\pi x}{a}\sin\frac{n\pi y}{b}$$

Substituting the solution and the load into the equations above yields [18] (10.2.9) (p. 368):

(A27) $${\hat{s}}_{11}W_{mn}+{\hat{s}}_{12}X_{mn}+{\hat{s}}_{13}Y_{mn}=Q_{mn}\;{\hat{s}}_{12}W_{mn}+{\hat{s}}_{22}X_{mn}+{\hat{s}}_{23}Y_{mn}=0\;{\hat{s}}_{13}W_{mn}+{\hat{s}}_{23}X_{mn}+{\hat{s}}_{33}Y_{mn}=0$$

where:

(A28) $${\hat{s}}_{11}=K_{s}\left(A_{55}{\alpha_{m}}^{2}+A_{44}{\beta_{n}}^{2}\right),\;{\hat{s}}_{12}=K_{s}A_{55}\alpha_{m},\;{\hat{s}}_{13}=K_{s}A_{44}\beta_{n}\;{\hat{s}}_{22}=D_{11}{\alpha_{m}}^{2}+D_{66}{\beta_{n}}^{2}+K_{s}A_{55},\;{\hat{s}}_{23}=\left(D_{12}+D_{66}\right)\alpha_{m}\beta_{n},\;{\hat{s}}_{33}=D_{66}{\alpha_{m}}^{2}+\;D_{22}{\beta_{n}}^{2}+K_{s}A_{44}$$

and $\alpha_{m}=m\pi /a$, $\beta_{n}=n\pi /b$.

Coefficients *b* are now defined:

(A29) $$b_{0}={\hat{s}}_{22}{\hat{s}}_{33}-{\hat{s}}_{23}{\hat{s}}_{23},\;b_{1}={\hat{s}}_{23}{\hat{s}}_{13}-{\hat{s}}_{12}{\hat{s}}_{33},\;b_{2}={\hat{s}}_{12}{\hat{s}}_{23}-{\hat{s}}_{22}{\hat{s}}_{13},\;b_{mn}={\hat{s}}_{11}b_{0}+{\hat{s}}_{12}b_{1}+{\hat{s}}_{13}b_{2}$$

Fourier coefficients are [18] (10.2.14) (p. 369):

(A30) $$W_{mn}=\frac{b_{0}}{b_{mn}}Q_{mn},\;X_{mn}=\frac{b_{1}}{b_{mn}}Q_{mn},\;Y_{mn}=\frac{b_{2}}{b_{mn}}Q_{mn}$$

This the end of Reddy’s text, the rest is a consequential result.

For an evenly distributed load $q\left(x,y\right)=q_{0}$, the coefficients $Q_{mn}$ are:

(A31) $$Q_{mn}=\frac{16q_{0}}{\pi^{2}mn}\;m,n=1,3,5,7\ldots$$

(A32) $$\mathrm{At}\;\mathrm{the}\;\mathrm{plate}’s\;\mathrm{mid}\text{-}\mathrm{point}\;\mathrm{at}\;\mathrm{location}\;x=\frac{a}{2};\;y=\frac{b}{2},\;\mathrm{exists}\;\sin\frac{m\pi x}{a}\sin\frac{n\pi y}{b}={\left(-1\right)}^{\frac{m+n}{2}-1}$$

The deflection at that point is the maximal deflection $w_{\max}$ which is:

(A33) $$w_{\max}=\sum_{n=1}^{\infty }\sum_{m=1}^{\infty }W_{mn}{\left(-1\right)}^{\frac{m+n}{2}-1}=\sum_{n=1}^{\infty }\sum_{m=1}^{\infty }\frac{b_{0}}{b_{mn}}Q_{mn}{\left(-1\right)}^{\frac{m+n}{2}-1}=\frac{16q_{0}}{\pi^{2}}\sum_{n=1,3,5\ldots }^{\infty }\sum_{m=1,3,5\ldots }^{\infty }\frac{b_{0}}{b_{mn}}\cdot \frac{{\left(-1\right)}^{\frac{m+n}{2}-1}}{mn}\;w_{\max}=\frac{16q_{0}}{\pi^{6}}\sum_{n=1,3,5\ldots }^{\infty }\sum_{m=1,3,5\ldots }^{\infty }\pi^{4}\frac{b_{0}}{b_{mn}}\cdot \frac{{\left(-1\right)}^{\frac{m+n}{2}-1}}{mn}$$

where

(A34) $$\frac{b_{0}}{b_{mn}}=\frac{{\hat{s}}_{22}{\hat{s}}_{33}-{\hat{s}}_{23}{\hat{s}}_{23}}{{\hat{s}}_{11}b_{0}+{\hat{s}}_{12}b_{1}+{\hat{s}}_{13}b_{2}}=\frac{{\hat{s}}_{22}{\hat{s}}_{33}-{\hat{s}}_{23}{\hat{s}}_{23}}{{\hat{s}}_{11}\left({\hat{s}}_{22}{\hat{s}}_{33}-{\hat{s}}_{23}{\hat{s}}_{23}\right)+{\hat{s}}_{12}\left({\hat{s}}_{23}{\hat{s}}_{13}-{\hat{s}}_{12}{\hat{s}}_{33}\right)+{\hat{s}}_{13}\left({\hat{s}}_{12}{\hat{s}}_{23}-{\hat{s}}_{22}{\hat{s}}_{13}\right)}$$

As in Libove’s solution above, Reddy’s solution (A33) presents the linear relationship between the load and the deflection in a small deflection case: $w_{max}=A\cdot q_{0}$.

### Appendix A.4. Simplified Libove’s Solutions

In multiwall plates, the *x*-direction shear rigidity is often very high: $S_{x}\to \infty$.

This causes the coefficients *K_i_* to change:

(A35) $$K_{1}=\frac{D_{xy}D_{x}}{2S_{y}};\;K_{2}=\frac{D_{x}D_{y}-D_{xy}D_{x}\mu_{y}}{S_{y}};\;K_{3}=\frac{D_{xy}D_{y}}{2S_{y}}\;K_{4}=0;\;K_{5}=-D_{x};\;K_{6}=-2\left[D_{xy}\left(1-\mu_{x}\mu_{y}\right)+D_{x}\mu_{y}\right];\;K_{7}=-D_{y}\;K_{8}=0;\;K_{9}=0;\;K_{10}=0\;\begin{array}{c} K_{11}=\frac{D_{xy}\left(1-\mu_{x}\mu_{y}\right)}{2S_{y}}\;;\;K_{12}=\frac{D_{y}}{S_{y}}\;;\;K_{13}=-\left(1-\mu_{x}\mu_{y}\right) \end{array}$$

This change simplifies the calculation a little bit:

(A36) $$\begin{array}{l} w_{max}=\frac{16q_{0}}{\pi^{6}}\sum_{n=1,3,5\ldots }^{\infty }\sum_{m=1,3,5\ldots }^{\infty }\frac{\left[-\pi^{2}K_{11}{\left(\frac{m}{a}\right)}^{2}-\pi^{2}K_{12}{\left(\frac{n}{b}\right)}^{2}+K_{13}\right]{\left(-1\right)}^{\frac{m+n}{2}-1}}{mn\left[\begin{array}{l} -\pi^{2}K_{1}{\left(\frac{m}{a}\right)}^{6}-\pi^{2}K_{2}{\left(\frac{m}{a}\right)}^{4}{\left(\frac{n}{b}\right)}^{2}-\pi^{2}K_{3}{\left(\frac{m}{a}\right)}^{2}{\left(\frac{n}{b}\right)}^{4}\\ +K_{5}{\left(\frac{m}{a}\right)}^{4}+K_{6}{\left(\frac{m}{a}\right)}^{2}{\left(\frac{n}{b}\right)}^{2}+K_{7}{\left(\frac{n}{b}\right)}^{4} \end{array}\right]} \end{array}$$

For orthotropic plates, which are rigid for shear deformation in both directions, (A36) reduces to:

(A37) $$w_{max}=\frac{16\left(\mu_{x}\mu_{y}-1\right)q_{0}}{\pi^{6}}\sum_{n=1,3,5\ldots }^{\infty }\sum_{m=1,3,5\ldots }^{\infty }\frac{{\left(-1\right)}^{\frac{m+n}{2}-1}}{mn\left[-D_{x}{\left(\frac{m}{a}\right)}^{4}-2\left[D_{xy}\left(1-\mu_{x}\mu_{y}\right)+D_{x}\mu_{y}\right]{\left(\frac{m}{a}\right)}^{2}{\left(\frac{n}{b}\right)}^{2}-D_{y}{\left(\frac{n}{b}\right)}^{4}\right]}$$

while, for isotropic plates, it further reduces to:

(A38) $$\begin{array}{c} w_{max}=\frac{16q_{0}b^{4}}{D\pi^{6}}\sum_{n=1,3,5\ldots }^{\infty }\sum_{m=1,3,5\ldots }^{\infty }\frac{{\left(-1\right)}^{\frac{m+n}{2}-1}}{mn{\left(m^{2}+{\left(\frac{b}{a}\right)}^{2}n^{2}\right)}^{2}} \end{array}$$

and for a square plate (*a* = *b*):

(A39) $$w_{max}=\frac{16q_{0}a^{4}}{D\pi^{6}}\sum_{n=1,3,5\ldots }^{\infty }\sum_{m=1,3,5\ldots }^{\infty }\frac{{\left(-1\right)}^{\frac{m+n}{2}-1}}{mn{\left(m^{2}+n^{2}\right)}^{2}}=0.0040624\frac{q_{0}a^{4}}{D}$$

## Appendix B

### Large Deflection of Thin Square Isotropic Plate with Distributed Load and Movable Edges

Several sources present information describing the large deflection of these plates. In order to compare the findings, it is necessary to normalize the results to a common format. Many articles present the following normalization:

(A40) $$q=Aw+Bw^{3}=N_{1}\frac{D}{a^{4}}w+N_{3}\frac{Eh}{a^{4}}w^{3}$$

Which leads to:

(A41) $${\frac{qa^{4}}{Eh^{4}}=N}_{1}\frac{1}{12\left(1-\nu^{2}\right)}\left(\frac{w}{h}\right)+N_{3}{\left(\frac{w}{h}\right)}^{3}$$

where the notations are described in the article above.

This normalization allows for a comparison of the results of any plate’s material-dimensions combination.

CPT: Note that the small deflection linear Classical Plate Theory (CPT) of Navier’s solution for this case, with large number of terms in the summation, has:

(A42) $$q=N_{1}\frac{D}{a^{4}}w=246.16\frac{D}{a^{4}}w\overset{yields}{\to }\frac{qa^{4}}{Eh^{4}}=\frac{246.16}{12\left(1-\nu^{2}\right)}\left(\frac{w}{h}\right)$$

Timoshenko [3] (p. 110) states the first summation term coefficient only: $N_{1}=240.38$.

Yankelevsky D. et al [19] (2017, Hebrew language) offer simple approximated solutions for three BCs, including the one stated here. For a single degree of freedom model SDOF, (p. 7), the plate deflection is:

(A43) $$q=389.64\frac{D}{a^{4}}w+6.3238\frac{Eh}{a^{4}}w^{3}$$

Walter D. Pilkey [20] (2005) p. 1001 offers an approximated solution for rectangular plate:

(A44) 16a4qπ6D=1+β22w0+3.88β21-ν2β2+0.6+1β2h2w03, β=ab

Then for a square plate:

(A45) $$q=240.35\frac{D}{a^{4}}w+7.4723\frac{Eh}{a^{4}}w^{3}$$

Levy Samuel [21] (1942), in NACA Report 737, solved the problem with Fourier series, creating an infinite system of non-linear algebraic equations to be solved. The truncation makes the solution an approximated one. He did that (manually!) for the first several terms for deflections up to 3.6 times the thickness. He also states that higher deflection requires more terms in order to converge accurately enough. The process is rather complex and is not easy to implement.

Using the data presented in [21] (Table 6 at p. 155), and in the graph at [21] (Figure 7 at p. 144) of the report, we have extracted the following load-deflection expression:

(A46) $$q=240.475\frac{D}{a^{4}}w+8.95617\frac{Eh}{a^{4}}w^{3}$$

Ishizaki Hatsuo [22] (1972) did many actual loading tests of flat glass deflections up to 10 times the thickness or breakage. The results were transferred to an Excel sheet for analysis, and the resulting expression is:

(A47) $$q=220\frac{D}{a^{4}}w+1.7787\frac{Eh}{a^{4}}w^{3}$$

The first number $N_{1}$ (small deflection coefficient) is reasonable, but the membrane coefficient $N_{3}$ is rather low, indicating a more flexible plate than in other research.

ASTM E 1300 [23] (2009) “Standard Practice for Determining Load Resistance of Glass in Buildings” supplies data for flat glass under load. Using the same thicknesses and glass properties as in Ishizaki above, as well as deflections up to five times the thickness, we get the following load expression:

(A48) $$q=243.2\frac{D}{a^{4}}w+2.29\frac{Eh}{a^{4}}w^{3}$$

Scholes A. [24] (1969) performed several actual tests that were said to agree well with other real tests done by Kaiser Rudolf (1936) and Stippes M. (1959). He tested 3.25 mm thick aluminum plate and deflections up to 3.3 times the thickness.

The expression found in his work is:

(A49) $$q=260.3\frac{D}{a^{4}}w+3.547\frac{Eh}{a^{4}}w^{3}$$

Kaiser Rudolf [25] (1936) performed an actual test on 600 × 600 × 3.15 mm steel plate up to 2.57 times the thickness. Although only one load–deflection point is declared, some intermediate points are implicitly given. After correcting the wrong reported load data with the supplied water manometer information, the following expression was calculated:

(A50) $$q=251.98\frac{D}{a^{4}}w+3.366\frac{Eh}{a^{4}}w^{3}$$

Chia Chuen-Yuan [26] (1980) (p. 64 expression 2-46, p. 65 expression 2-50) is similar to Levy [21], using the first term only (a) and eight terms (b), and shows expressions, for this case, with deflections up to two times the thickness:

(A51) $$(a)\;q=240.35\frac{D}{a^{4}}w+3.7972\frac{Eh}{a^{4}}w^{3}$$

(A52) $$(b)\;q=240.35\frac{D}{a^{4}}w_{0}+3.9008\frac{Eh}{a^{4}}{w_{0}}^{3}$$

Brown, J.C. [27] (1969) performed several actual experiments with rectangular aluminum plates of 0.81, 1.02, 1.29, and 1.63 mm thickness, with deflections up to 4.6 times the thickness. The reported results were converted to the standard form:

(A53) $$q=307.9\frac{D}{a^{4}}w+2.799\frac{Eh}{a^{4}}w^{3}$$

Present study: to complete this comparison, the expression found in the present study is:

(A54) $$q=246.16\frac{D}{a^{4}}w_{0}+18.6\sqrt{\frac{h}{a}}\cdot \frac{Eh}{a^{4}}{w_{0}}^{3}$$

Table A1 summarizes the coefficients found by the various authors and presented in the literature.

**Table A1:** Summary of the N_1_ and N_3_ coefficients found in the literature.

No.	Ref.	Source	$N_{1}$	$N_{3}$
1	[3]	Navier’s linear CPT	246.16	-
2	[19]	Yankelevsky D. et al.	389.64	6.3238
3	[20]	Walter D. Pilkey	240.35	7.4723
4	[21]	Levy Samuel	240.475	8.9562
5	[22]	Ishizaki Hatsuo	220	1.7787
6	[23]	ASTM E 1300	243.2	2.29
7	[24]	Scholes A.	260.3	3.547
8	[25]	Kaiser Rudolf	251.98	3.366
9	[26]	Chia Chuen-Yuan	240.35	3.7972
10	[27]	Brown J. C.	307.9	2.799
11	-	Present study-for *h*/*a* = 0.025	246.16	2.94

It is obvious that, although most of data is based on actual experiments, a considerable variability exists here, especially in the large deflection coefficient $N_{3}$. The $N_{3}$ variability may be explained with the suggested *h*/*a* ratio. This should be further checked for other possible explanations.

## Appendix C

### Plates Properties, Loading Test and FEA Data

**Table A2:** Properties of plates used in vacuum chamber tests.

			Polygal	Polygal	Polygal	Polygal	Faculty Lab
Thickness	*h*	[mm]	6	8	10	16	10
Area Weight	*W*	[g/m²]	1305	1492	1683	2712	1713
Walls Thickness	*t*_w_	[mm]	0.76	0.981	1.055	1.745	1.154
Equivalent Gt	*G*_t_^eq^	[MPa]	110.14	106.63	91.739	94.837	100.35
Equivalent Gb	*G*_b_^eq^	[MPa]	157.61	135.14	121.96	122.83	124.13
	For small deflection coefficient *A*:						
	*D_x_*	[Nm]	18.438	40.896	70.421	234.91	70.121
Bending:	*D_y_*	[Nm]	14.374	38.000	58.298	205.04	54.104
	*D_xy_*	[Nm]	2.8370	5.7662	10.163	41.925	10.344
	*S_x_*	[N/m]	10,272	51,480	70,445	244,549	59,890
	*S_y_*	[N/m]	3956.9	2201.8	2365.4	3335.3	1662.1
	*ν_x_* ^b^		0.38	0.38	0.38	0.38	0.38
	*ν_y_*^b^ = *D_y_*/*D_x_* ∗ *ν_x_*^b^		0.296	0.353	0.315	0.332	0.293
	For large deflection coefficient *B*:						
			6/1300	8/1500	10/1700	16/2700	10/1700
	*E_x_*	[MPa]	435.00	373.00	336.60	339.00	342.60
Tension:	*E_y_*	[MPa]	304.00	294.30	253.20	261.75	276.96
	*ν_x_* ^t^		0.38	0.38	0.38	0.38	0.38
	*ν_y_*^t^ = *E_y_*/*E_x_* ∗ *ν_x_*^t^		0.266	0.300	0.286	0.293	0.307

**Table A3:** Properties of plates used in the FEA.

Thickness	*h*	[mm]	6	8	10	16
Area Weight	*W*	[g/m²]	1300	1500	1700	2700
Walls Thickness	*t*_w_	[mm]	0.76	0.981	1.055	1.745
Equivalent Gt	*G*_t_^eq^	[MPa]	110.14	106.63	91.739	94.837
Equivalent Gb	*G*_b_^eq^	[MPa]	157.00	135.87	123.19	122.28
	For small deflection coefficient *A*:					
	*D_x_*	[Nm]	16.3625	35.4086	62.0025	205.3052
Bending:	*D_y_*	[Nm]	12.7562	32.9011	51.3292	179.2000
	*D_xy_*	[Nm]	2.8261	5.7971	10.2657	41.7391
	*S_x_*	[N/m]	288,060	230,560	294,700	407,840
	*S_y_*	[N/m]	3840	2240	2400	3200
	*ν_x_* ^b^		0.38	0.38	0.38	0.38
	*ν_y_*^b^ = *D_y_*/*D_x_* ∗ *ν_x_*^b^		0.296		0.353	0.315
	For large deflection coefficient *B*:					
			6/1300	8/1500	10/1700	16/2700
	*E_x_*	[MPa]	433.33	375.00	340.00	337.50
Tension:	*E_y_*	[MPa]	304.00	294.30	253.20	261.75
	*ν_x_* ^t^		0.38	0.38	0.38	0.38
	*ν_y_*^t^ = *E_y_*/*E_x_* ∗ *ν_x_*^t^		0.266	0.267	0.298	0.283

In the following tables, the unit of width/length is [m], the unit of *A* is [Pa/m], and the unit of *B* is [Pa/m³].

**Table A4:** The 6 mm plate Polygal vacuum tests *A*, *B* and theory calculated *A*, *B*.

Width [m]	Length [m]	*A*	*B*	Cal *A*	Cal *B*
1.12	2.32	452.9958	2,675,924.5	838.0078	235,076.9
1.12	1.92	600.2268	3,624,304.7	947.2736	423,544.7
1.12	1.42	6495.11	3,478,986.3	1340.64	1001,306
1.12	1.17	6866.385	5,022,146.1	1892.093	1,650,043
1.12	0.92	8989.318	9,007,183.6	3341.16	2,892,938
0.97	2.32	1805.044	689,141.94	1394.157	280,299.7
0.97	1.92	3589.357	1,571,819.9	1510.433	518,885.1
0.97	1.42	9345.895	2,682,268.9	1947.39	1,284,722
0.97	1.17	11,092.35	5,344,922.6	2553.413	2,183,605
0.97	0.92	14,659.1	9,617,604.9	4106.67	3,979,952
0.9	2.32	2846.633	1,042,440.4	1833.756	304,838
0.9	1.92	3992.159	2,319,479.3	1950.029	572,014.2
0.9	1.42	8516.378	6,275,772.6	2409.381	1,449,841
0.9	1.17	9318.191	7,315,412.6	3048.365	2,503,720
0.9	0.92	30,982.47	7,447,190.6	4668.252	4,656,408
0.72	2.32	7953.529	2,283,356.5	4250.167	380,062.2
0.72	1.92	9081.767	3,334,754.5	4334.273	740,756.6
0.72	1.42	15,608.04	6,558,901	4830.433	2006,872
0.72	1.17	17,281.05	10,784,310	5576.681	3628,605
0.72	0.92	21,693.31	23,693,730	7445.51	7,160,451
0.72	0.72	30,733.39	32,097,457	11,636.92	13,408,479
0.52	2.32	17,991.24	4,017,196	14,732.46	488,196
0.52	1.92	19,992.22	7,342,052.5	14,648.92	998,573.1
0.52	1.67	18,881.28	12,322,531	14,669.48	1,663,546
0.52	1.42	20,924.37	34,600,895	14,902.81	2,951,309
0.52	1.17	31,266.07	32,159,471	15,684.94	5,676,341
0.52	0.92	39,697.58	44,985,911	17,947.36	12,157,585
0.52	0.72	41,093.97	110,628,017	23,031.12	24,851,580

**Table A5:** The 8 mm plate Polygal vacuum tests *A*, *B* and theory calculated *A*, *B*.

Width [m]	Length [m]	*A*	*B*	Cal *A*	Cal *B*
1.12	2.32	10,615.63	1,714,038	1998.142	326,809.6
1.12	1.92	9658.971	3,702,746	2240.471	588,822
1.12	1.67	12,600.17	3,940,898	2546.095	887,351.3
1.12	1.42	12,449.55	5,276,544	3126.229	1,392,040
1.12	1.17	11,817.76	6,953,640	4378.486	2,293,929
1.12	0.92	14,593.63	11,593,336	7702.517	4,021,832
1.12	0.72	17,232.15	13,744,086	15,946.62	6,673,482
0.97	2.32	12,245.53	2,858,078	3233.376	389,679.3
0.97	1.92	12,727.31	6,404,959	3482.44	721,366.5
0.97	1.67	16,119.69	7,413,828	3812.679	1,110,141
0.97	1.42	18,888.9	12,222,746	4445.654	1,786,052
0.97	1.17	18,311.34	15,101,302	5799.834	3,035,700
0.97	0.92	23,887.15	22,780,661	9315.615	5,533,025
0.97	0.72	30,987.88	27,779,780	17,806.58	9,546,048
0.9	2.32	12,629.23	2,398,253	4174.936	423,793
0.9	1.92	8274.929	3,639,945	4417.666	795,227.9
0.9	1.67	8759.203	4,771,837	4756.228	1,236,838
0.9	1.42	17,964.97	8,229,842	5415.468	2,015,603
0.9	1.17	12,983.75	14,426,740	6827.399	3,480,732
0.9	0.92	18,981.43	17,899,379	10,459.87	6,473,451
0.9	0.72	24,429.51	35,284,855	19,115.65	11,400,203
0.72	2.32	22,097.52	3,652,679	8994.497	528,371.7
0.72	1.92	24,121.8	4,877,503	9143.279	1,029,818
0.72	1.67	14,853.55	15,352,886	9456.951	1,650,391
0.72	1.42	16,587.32	10,589,949	10,156.51	2,790,001
0.72	1.17	15,934.62	8,425,231	1,1731.1	5,044,573
0.72	0.92	21,440.99	29,554,347	15,759.63	9,954,631
0.72	0.72	21,748.56	33,151,357	25,043.26	18,640,790

**Table A6:** The 10 mm plate Polygal vacuum tests *A*, *B* and theory calculated *A*, *B*.

Width [m]	Length [m]	*A*	*B*	Cal *A*	Cal *B*
1.12	2.32	5279.765	892,109.2	2897.421	403,685.8
1.12	1.92	6410.03	1,607,430	3286.038	727,332
1.12	1.67	5226.576	4,155,473	3775.575	1,096,085
1.12	1.42	10,148.55	4,496,982	4709.153	1,719,493
1.12	1.17	14,394.48	6,498,713	6742.018	2,833,536
1.12	0.92	19,032.32	11,718,453	12,205.65	4,967,897
1.12	0.72	28,726.07	21,991,590	25,938.38	8,243,301
0.97	2.32	5008.443	1,513,391	4596.355	481,344.5
0.97	1.92	7734.193	1,955,618	4997.688	891,055.2
0.97	1.67	7097.357	4,643,788	5523.879	1371,281
0.97	1.42	12,476.48	5,000,107	6532.75	2,206,189
0.97	1.17	14,198.68	10,234,183	8706.231	3,749,795
0.97	0.92	20,930.16	15,246,556	14,421.05	6,834,571
0.97	0.72	37,527.73	19,321,951	28,430.69	11,791,587
0.9	2.32	8175.153	1,308,174	5870.023	523,482.9
0.9	1.92	9144.266	2,979,196	6264.635	982,291.3
0.9	1.67	13,540.09	2,623,564	6804.076	1,527,783
0.9	1.42	14,152.08	4,766,794	7850.599	2,489,738
0.9	1.17	11,179.3	16,477,742	10,103.28	4,299,513
0.9	0.92	20,422.31	43,633,063	15,970.46	7,996,216
0.9	0.72	27,507.29	48,216,872	30,171.04	14,081,899
0.72	2.32	9764.093	1,210,446	12,210.72	652,661.8
0.72	1.92	11,121.45	2,057,851	12,480.63	1,272,064
0.72	1.67	11,484.15	3,560,990	12,994.57	2,038,616
0.72	1.42	15,098.11	3,883,557	14,105.48	3,446,300
0.72	1.17	15,530.53	10,511,290	16,582.48	6,231,220
0.72	0.92	28,161.24	16,177,794	22,962.68	12,296,282
0.72	0.72	21,112.22	91,644,642	37,902.26	23,025,708
0.52	2.32	19,650.88	1,438,809	34,293.65	838,354.4
0.52	1.92	18,181.98	4,628,025	33,972.7	1,714,799
0.52	1.67	22,336.73	5,604,267	34,048.95	2,856,725
0.52	1.42	21,800.6	8,182,871	34,814.92	5,068,134
0.52	1.17	27,381.58	14,058,050	37,255.82	9,747,694
0.52	0.92	43,811.35	19,654,856	44,249.42	20,877,610
0.52	0.72	40,750.83	78,821,611	60,519.49	42,676,370

**Table A7:** The 16 mm plate Polygal vacuum tests *A*, *B* and theory calculated *A*, *B*.

Width [m]	Length [m]	*A*	*B*	Cal *A*	Cal *B*
1.12	2.32	8933.279	1,421,073	8242.064	832,170.8
1.12	1.92	9052.37	1,657,316	9467.222	1499,345
1.12	1.42	12,481.09	6,361,606	13,963.99	3,544,618
1.12	1.17	16,163.43	11,237,602	20,463.26	5,841,141
1.12	0.92	35,908.31	13,121,229	38,217.02	10,240,980
0.97	2.32	9890.783	1,140,765	12,333.83	992,258.9
0.97	1.92	10,953.37	2,569,300	13,586.46	1,836,850
0.97	1.42	16,100.85	7,337,956	18,328.21	4,547,908
0.97	1.17	21,039.46	11,380,187	25,112.24	7,729,946
0.97	0.92	42,337.42	16,439,904	43,280.84	14,089,003
0.9	2.32	13,151.49	1,481,442	15,239.14	1,079,124
0.9	1.92	17,183.46	2,547,259	16,475.18	2,024,927
0.9	1.42	21,875.81	11,601,028	21,323.35	5,132,426
0.9	1.17	32,690.09	21,102,044	28,261.72	8,863,153
0.9	0.92	48,325.19	27,733,684	46,679.89	16,483,654
0.72	2.32	21,365.54	1614,211	28,516.63	1,345,418
0.72	1.92	19,605.54	3,484,898	29,481.13	2,622,273
0.72	1.42	27,959.72	8239,599	34,419.8	7,104,312
0.72	1.17	32,710.82	14,309,190	41,783.57	12,845,236
0.72	0.92	48,831.18	21,922,037	61,033.98	25,347,948
0.52	2.32	40,168.46	122,204.1	68,615.16	1,728,210
0.52	1.92	39,558.79	12,311,136	68,271.49	3,534,942
0.52	1.42	34,807.32	18,890,827	71,902.62	10,447,613
0.52	0.92	56,908.98	48,134,886	99,778.62	4,3037,770

**Table A8:** The 10 mm plate faculty lab vacuum tests *A*, *B* and theory calculated *A*, *B*.

Width [m]	Length [m]	*A*	*B*	Cal *A*	Cal B
1.13	2.33	3658.214	648,934.7	2479.251	413,461.1
0.73	1.43	9602.007	5,370,164	11,487.23	3,475,817
1.43	0.73	19,061.98	5,858,385	21,805.59	4,378,295
0.73	0.73	27,613.32	36,089,724	32,835.42	22,731,859

**Table A9:** The 6 mm plate FEA *A*, *B* and theory calculated *A*, *B*.

Width [m]	Length [m]	*A*	*B*	Cal *A*	Cal *B*
1.2	2.4	1352.5	195,200.78	583.00495	192,742.6
1.2	2	1690.6	342,476.002	664.74488	337,874.6
1.2	1.5	2435.8	771,141.611	943.8827	762,498.9
1.2	1	3780.8	2,096,942.96	2237.456	2,054,472
1.2	0.8	5459.8	3,230,529.03	4304.5028	3,277,946
1	2.4	2020.9	237,038.006	1106.511	241,597.5
1	2	2427.3	432,414.367	1196.5063	437,817.7
1	1.5	3415.9	1,046,885.37	1517.8621	1,044,852
1	1	5436.3	3,136,751.28	2956.0945	3,057,565
1	0.8	7312.3	5,186,080.43	5156.837	5,104,099
0.8	2.4	3697.7	286,277.87	2538.5252	305,448.5
0.8	2	4208	537,577.951	2619.1872	574,768.8
0.8	1.5	5508.9	1,396,552.89	2984.2191	1,463,440
0.8	1	8723.5	4,727,916.95	4665.1049	4,727,700
0.8	0.8	11,347	8,469,456.9	7125.0737	8,345,855
0.6	2.4	7826.7	440,304.645	7656.8973	388,706.2
0.6	2	8547.9	767,570.33	7650.9892	763,622.2
0.6	1.5	10,104	2,014,716.54	7949.0107	2,097,575
0.6	1	14,283	7,744,919.53	9969.4304	7645,229
0.6	0.8	18,211	15,310,356.5	12,938.884	14,487,347

**Table A10:** The 8 mm plate FEA *A*, *B* and theory calculated *A*, *B*.

Width [m]	Length [m]	*A*	*B*	Cal *A*	Cal *B*
1.2	2.4	2831.98	209,943	1391.957	268,758.5
1.2	2	3318.587	382,338.8	1571.233	471,129.2
1.2	1.5	4829.209	836,190.2	2188.248	1,063,221
1.2	1	8415.004	2,126,931	5050.915	2,864,736
1.2	0.8	12,239.55	3,542,515	9634.085	4,570,738
1	2.4	3826.083	282,870.4	2576.69	336,881.3
1	2	4546.522	509,018.9	2766.492	610,488.9
1	1.5	6349.96	1,214,195	3460.85	1,456,932
1	1	10,850.79	3,487,875	6598.992	4,263,441
1	0.8	15,045.61	6,025,413	11,425.3	7,117,108
0.8	2.4	6466.841	378,227.5	5618.453	425,914.5
0.8	2	7303.275	683,105.1	5771.521	801,452.3
0.8	1.5	9412.111	1,737,764	6521.063	2,040,607
0.8	1	15,196.44	5,805,936	10,074.37	6,592,261
0.8	0.8	20,606.35	10,550,171	15,335.99	11,637,383
0.6	2.4	13,854.28	637,013.4	15,284.07	542,008.4
0.6	2	14,604.9	1,072,049	15,231.35	1,064,788
0.6	1.5	16,662.53	2,699,289	15,760.38	2,924,839
0.6	1	22,717.44	10,523,348	19,741.63	10,660,436
0.6	0.8	29,149.22	21,212,249	25,735.51	20,201,022

**Table A11:** The 10 mm plate FEA *A*, *B* and theory calculated *A*, *B*.

Width [m]	Length [m]	*A*	*B*	Cal *A*	Cal *B*
1.2	2.4	3471.776	266,698.2	2074.733	332,895.2
1.2	2	4277.692	464,471	2367.607	583,559.8
1.2	1.5	6167.916	1,034,814	3378.928	1,316,949
1.2	1	11,004.35	2,840,307	8155.409	3,548,379
1.2	0.8	16,640	5,287,136	15,925.41	5,661,502
1	2.4	5041.094	342,169.1	3752.39	417,274.8
1	2	5969.138	613,404.1	4063.639	756,176.4
1	1.5	8251.718	1,477,673	5190.444	1,804,615
1	1	14,203.62	4,449,369	10,353.93	5,280,871
1	0.8	20,480.89	8,169,863	18,434.41	8,815,540
0.8	2.4	8484.767	470,300.6	7924.851	527,555.1
0.8	2	9522.598	841,360.8	8188.214	992,711.5
0.8	1.5	12,163.73	2,145,383	9403.726	2,527,578
0.8	1	19,432.33	7,450,490	15,143.81	8,165,442
0.8	0.8	26,721.05	14,272,233	23,786.57	14,414,536
0.6	2.4	18,260.27	779,447.6	20,585.35	671,353.6
0.6	2	19,010.76	1,319,853	20,543.94	1,318,889
0.6	1.5	21,417.6	3,330,184	21,477.9	3,622,825
0.6	1	29,186.41	13,096,619	27,827.71	13,204,450
0.6	0.8	38,296.02	26,541,955	37,407.44	25,021,808

**Table A12:** The 16 mm plate FEA *A*, *B* and theory calculated *A*, *B*.

Width [m]	Length [m]	*A*	*B*	Cal *A*	Cal *B*
1.2	2.4	7300.703	539,625.82	6043.828	680,603.8
1.2	2	8756.195	962,335.5	6968.5	1,193,087
1.2	1.5	12,494.57	2,256,154.1	10,164.26	2,692,501
1.2	1	25,073.1	7,181,867.8	25,486.15	7,254,656
1.2	0.8	48,174.93	10,714,430	50,808.47	11,574,934
1	2.4	10,234.71	754,218.1	10,192.25	853,117.9
1	2	11,604.14	1,394,012.3	11,158.47	1,546,002
1	1.5	15,309.64	3,595,719.6	14,616.61	3,689,533
1	1	29,275.91	11,815,887	30,707.89	10,796,736
1	0.8	52,687.53	18,995,236	56,414.56	18,023,361
0.8	2.4	18,117.44	910,655.82	19,449.97	1,078,586
0.8	2	19,822.02	1,640,282.7	20,303.23	2,029,597
0.8	1.5	24,555.24	4,306,897.9	23,945.99	5,167,631
0.8	1	40,674.54	15,820,108	41,107.7	16,694,237
0.8	0.8	64,001.02	29,188,219	67,574.55	29,470,502
0.6	2.4	37,083.77	1,376,602.7	43,775.8	1,372,582
0.6	2	37,921.72	2,428,218.6	43,952.9	2,696,468
0.6	1.5	41,970.62	6,360,732.5	47,090.34	7406,861
0.6	1	59,121.26	25,612,031	65,386.7	26,996,483
0.6	0.8	83,851.03	52,164,977	93,201.39	51,157,057

## Appendix D

### Appendix D.1. FEA Description

#### Appendix D.1.1. The Analysis

The FEA (Finite Element Analysis) software is the Siemens Simcenter Femap with Nastran Ver. 2021.1. The FEA process has three basic steps: model preparation, running the solver, and post-processing.

#### Appendix D.1.2. The Model Preparation

To get correct results, we use a consistent system of units:

- Length-mm
- Force-N
- Stress, Elastic Modulus-MPa [N/(mm)²]
- Shear Rigidity-N/mm

#### Appendix D.1.3. Materials and Property

In Femap, the 2D plate element Property allows for the definition of a different material for every one of the four elastic response modes:

- In-plane tension/compression and shear
- Out-of-plane bending and twist
- Cross-section transverse shear deformation
- Coupling of membrane-bending due to asymmetric structure

This ability is suitable to correctly represent the multiwall plate equivalent elastic constants. In our case, however, since the multiwall plate is orthotropic symmetric, the last coupling material is not necessary, and therefore, it is left ignored.

We define three 2D Orthotropic Materials: TensionMat, BendingMat, ShearMat, and we fill their elastic moduli fields with the values found before (see Appendix C).

### Appendix D.2. Geometry, Boundary Conditions (BC), Load

The geometry in use is rectangular surfaces, length in *x*-direction, and width in *y*-direction. The edges BCs (constraints) are simply supported, in-plane movable on all edges, and designated SSSS-M. In Femap, these constraints are designated “3” for *z* translation only.

The plate is loaded with an evenly distributed load realized as pressure on the surface. This pressure load is operating perpendicular to the surface, which means that it slightly changes its direction while the plate deflects, i.e., it is a follower force. This type of load is best used to simulate wind load or vacuum chamber test.

### Appendix D.3. Meshing

Next, we mesh the model with the Plate Property. A relatively small number of elements is enough here, as the equivalent element only considers the major response, neglecting all of the plate’s small features. A 50 × 50mm element was used, resulting in various numbers of square Quad 4-noded elements. The default direction of the orthotropic element *x*-direction is naturally *x*, so no explicit definition of the orientation is necessary.

### Appendix D.4. More BCs after Meshing

The analysis program requires that all rigid body degrees of freedom (DOFs) will be eliminated. Therefore, virtual BCs (not participating in the analysis) should be added to cancel these DOFs. The plate midpoint node gets 12 (no translation in *x*,*y*). One plate edge midpoint also gets a constraint that eliminates the free rigid body *z* rotation around the plate’s midpoint and also eliminates *z* deflection (13 or 23).

### Appendix D.5. Running the Solver

Large deflections with a non-linear response are expected, so it is necessary to use the non-linear static solution method. The solver uses the NX-Nastran SOL-106 routine.

### Appendix D.6. Post-Processing

Femap allows us to obtain the load–deflection graphs at the requested nodes. The graph data are then transferred to Excel, which produces graphs as shown in Figure 3. The Excel sheet also performs theoretical least-squares regression analyses to find the coefficients *A* and *B* for each plate.

Figure A1 below shows a typical FEA screen where the mesh, BCs, loads, and deflections are presented.
